# Natural variation at *qHd1* affects heading date acceleration at high temperatures with pleiotropism for yield traits in rice

**DOI:** 10.1186/s12870-018-1330-5

**Published:** 2018-06-07

**Authors:** Jun-Yu Chen, Hong-Wei Zhang, Hua-Li Zhang, Jie-Zheng Ying, Liang-Yong Ma, Jie-Yun Zhuang

**Affiliations:** State Key Laboratory of Rice Biology and Chinese National Center for Rice Improvement, China National Rice Research Institute, Hangzhou, 310006 China

**Keywords:** Rice, Temperature response, Heading date, Grain filling, Adaption, Quantitative trait locus

## Abstract

**Background:**

Rice is highly sensitive to temperature fluctuations. Recently, the frequent occurrence of high temperature stress has heavily influenced rice production. Proper heading date in specific environmental conditions could ensure high grain yield. Rice heading greatly depends on the accurate measurement of environmental changes, particularly in day length and temperature. In contrary to the detailed understanding of the photoperiod pathway, little has been known about how temperature regulates the genetic control of rice heading.

**Results:**

Near isogenic lines that were segregated for *qHd1*, were developed from a cross between *indica* rice varieties Zhenshan 97 (ZS97) and Milyang 46 (MY46). Using a five sowing-date experiment in the paddy field, we observed the involvement of *qHd1* in temperature responses. With the gradual increase of temperature from Trial I to V, heading date of MY46 homozygotes continued to decrease for about 5 d per trial from 76 to 58 d, while that of ZS97 homozygotes was promoted at the same rate from Trial I to III and then stabilized at 69 d. This thermal response was confirmed in a temperature-gradient experiment conducted in the phytotron. It is also found that tolerance of the ZS97 allele to heading acceleration at high temperature was associated with higher grain weight that resulted in higher grain yield. Then, by qRT-PCR and RNA-seq, we found the pathway *OsMADS51-Ehd1-RFT1/Hd3a* underlying the *qHd1*-mediated floral response to temperature. By sequence comparison, *OsMADS51* for *qHd1* displayed a 9.5-kb insertion in the 1st intron of the ZS97 allele compared to the MY46 allele. Furthermore, this large insertion is commonly found in major early-season *indica* rice varieties, but not in the middle- and late-season ones, which corresponds to the requirement for high-temperature tolerance during the heading and grain-filling stages of early-season rice.

**Conclusions:**

Beneficial alleles at *qHd1* confer tolerance to high temperatures at the heading and grain-filling stages, playing a significant role in the eco-geographical adaptation of early-season *indica* rice during modern breeding. These results, together with the underlying *OsMADS51-Ehd1-RFT1/Hd3a* floral pathway, provide valuable information for better understanding the molecular mechanism of temperature responsive regulation of heading date and yield traits in rice.

**Electronic supplementary material:**

The online version of this article (10.1186/s12870-018-1330-5) contains supplementary material, which is available to authorized users.

## Background

In recent decades, the influence of climate changes on rice productivity has become increasingly concerned, such as more frequent high temperature stress accompanied with global warming [1]. A small increase in the daily mean temperature of near 2 °C would make a dramatic decrease in grain yield [2]; likewise, yield reduction could occur by 1 °C increase in the mean minimum temperature [3]. In rice production and breeding practice, heading date and yield traits are generally considered as key indicators for the response to temperature variation; for instance, early heading and yield loss have been commonly observed at high temperatures. Rice growth process consists of three sequential stages, i.e., vegetative, reproductive, and grain filling [4]. Flower initiation is the indication of transition from vegetative to reproductive stage, which largely determines the heading date variation among different rice genotypes and is governed by thermal sensitivity besides the basic vegetative growth and photoperiod sensitivity [5–7]. Poor seed setting and insufficient grain filling that result in yield loss are typical symptoms at high temperature during the second half of rice growth [8–10]. That is to say, rice is highly sensitive to temperature changes, particularly at the heading and grain-filling stages. To ensure proper seasonal and regional adaptation of rice varieties, heading date has always been a primary target in rice breeding. Clearly, understanding how temperature plays a role in regulating rice heading would be essential to mitigate the impacts of climate changes, particularly when temperature appears to be rising.

Unfortunately, neither gene cloning nor quantitative trait locus (QTL) primary mapping in rice has explored the genetic contributions of the thermo-sensitivity to heading date. Gene cloning exclusively focused on the photoperiodic regulation of heading and identified two independent gene pathways [11, 12]. One is the *Heading date 1* (*Hd1*) *–Heading date 3a* (*Hd3a*) pathway that is evolutionarily correlated with the *Arabidopsis CONSTANS* (*CO*) *–FLOWERING LOCUS T* (*FT*) pathway. The other is the *Early heading date 1* (*Ehd1*) *–Hd3a/ Rice flowering locus T1* (*RFT1*) pathway that is unique to rice. QTL primary mapping dealt with various aspects associated with heading date, including basic vegetative phase, photoperiod sensitivity, vegetative growth time, reproductive growth time, and days to maturity (http://archive.gramene.org/qtl/), but thermal sensitivity has not been taken into account. Therefore, it is urgent to identify genetic factors determining the response of rice heading to temperature variations. In *Arabidopsis thaliana*, studies have revealed the existence of at least some interactions between photoperiod and temperature pathway. For instance, *PHYTOCHROME INTERACTING FACTOR 4* could not display the thermal induction of flowering under continuous light, but this function was observed in short-day conditions [13]; a mutation at *EARLY FLOWERING 3* greatly influenced the sensitivity to temperature under abnormal photoperiod pathway [14]. Other genes associated with the response to both photoperiod and temperature changes included photoreceptors such as *CRYPTOCHROME 1* [15], *CRYPTOCHROME 2* [16] and *PHYTOCHROME B* [17, 18]. These findings provide evidences for the importance of thermo-sensitivity in regulating flowering in plants and offer reference to identify relevant molecular contributors in rice.

Using near isogenic lines (NILs) and NIL-F_2_ populations derived from a cross between *indica* rice varieties Zhenshan 97 (ZS97) and Milyang 46 (MY46), we previously fine-mapped *qHd1*, a minor-effect QTL for heading date independent of day-length [19]. Surprisingly, the effect of *qHd1* was greatly decreased in a following-up test in the winter rice-growing season in Lingshui, Hainan, China. Heading date difference between NILs *qHd1*^ZS97^ and *qHd1*^MY46^ was only 2.6 d (Additional file 1: Figure S1), much smaller than the difference of 5.3 d observed previously [19]. Therefore, we carried out the present study to investigate the relationship between the function of *qHd1* and temperature variation. We firstly conducted a multiple sowing-date experiment in the paddy field to test *qHd1*, followed by a validation in the controlled environment. Results suggest that *qHd1* regulates rice heading in response to temperature, and contributes to high-temperature tolerance at the heading and grain-filling stages. Then, by gene sequencing and expression analysis including RNA-seq, the temperature sensing pathway of *OsMADS51*-*Ehd1-Hd3a/RFT1* was identified, with gene *OsMADS51* underlying QTL *qHd1*. In addition, by genotyping 109 major *indica* rice varieties released in China since 1950s, evidence was shown for the significance of *qHd1* in the adaptation of early-season *indica* rice to high temperature during rice heading and grain filling. This information would be greatly useful for exploring the molecular mechanism of temperature-related regulation of rice heading in breeding practice.

## Methods

### Rice materials

Two sets of NILs and 109 *indica* rice varieties were used. The two NIL sets were segregated for *qHd1*, a minor-effect QTL for heading date [19]. They were derived from a BC_2_F_8_ plant of the *indica* rice cross ZS97///ZS97//ZS97/MY46 and named CJ1 and CJ2 (Additional file 2: Figure S2). Each NIL set contained 50 homozygous ZS97 lines and 50 homozygous MY46 lines. The 109 *indica* rice varieties consisted of three collections (Additional file 3: Table S1). The first collection contained 13 early-season varieties that had large planting areas for a long period before 1985. The second collection included 48 early-season, 24 middle-season and 10 late-season varieties that were planted in large areas for at least one year during 1990−2014 [20, 21]. The third collection had 14 early-season varieties, including Jiayu 253, Zhongxuan 181, and 12 descendants of either Jiayu 253 or Zhongxuan 181, which were released after 2000.

### Field experiments and trait evaluation

The two NIL sets were tested in the paddy field at the China National Rice Research Institute in Hangzhou, China. They were grown from April to October in 2015 with five sowing dates. The sowing and transplanting dates were 28 April and 25 May for Trial I, 8 May and 2 June for Trial II, 20 May and 11 June for Trial III, 15 June and 6 July for Trial IV, and 9 July and 29 July for Trial V, respectively (Table 1). A randomized complete block design with two replications was used. In each replication, each line was grown in a single row of 12 plants, with spacing of 16.7 cm between plants and 26.7 cm between rows. Field management followed the normal agricultural practice. The soil type belongs to purplish clayey soil and irrigation was applied to maintain a proper-watered condition from sowing to harvesting. During the period of floral transition in the rice materials tested, day length in Hangzhou was longer than 13 h (www.timeanddate.com), corresponding to natural long day (NLD) conditions [22–24]. From the weather station located near the experimental field, the data of daily mean temperature were also collected.

**Table 1:** The multiple sowing-date experiment conducted in Hangzhou in 2015

Trial	Sowing date	Transplanting date	Traits measured
I	28 April	25 May	HD, yield traits
II	8 May	2 June	HD
III	20 May	11 June	HD, yield traits
IV	15 June	6 July	HD, yield traits
V	9 July	29 July	HD

*HD* heading date; yield traits measured included number of panicle per plant (NP), number of grains per panicle (NGP), 1000-grain weight (TGW, g), spikelet fertility (SF, %) and grain yield per plant (GY, g)

Heading date (HD) was recorded for all the five trials, and yield traits were measured for Trial I, III and IV. HD was recorded for each plant when a panicle emerged. The data were averaged over all plants of each line in each replication. At maturity, five middle plants of each line were harvested in bulk and sun-dried. Five yield traits were measured, including number of panicles per plant (NP), number of grains per panicle (NGP), 1000-grain weight (TGW, g), spikelet fertility rate (SF, %), and grain yield per plant (GY, g). For measuring TGW, fully filled grains maintaining a constant condition among different samples were used as described previously [25].

### Phytotron experiment with temperature-gradient treatment

To validate the responsiveness of *qHd1* to temperature variation, NILs derived from CJ1 and consisted of two homozygous genotypes were grown in phytotrons at humidity of 70% with three different temperature treatments under short day (SD) conditions. The temperature-gradient test consisted of daily cycles of 11 h light at 26, 28 and 30 °C and 13 h dark at 22, 24 and 26 °C, respectively. Two lines were planted for each genotype, with eight plants per line. Heading date was scored for each plant.

### Quantitative real-time PCR (qRT-PCR) analysis

Penultimate leaves of the two homozygous genotypes of CJ1 were collected at 8:30–9:00 am in 30, 35 and 45 days after sowing with the same sowing date to Trial IV, well covering the transition period from vegetative to reproductive phase. One sample consisted of three leaves and three samples were analyzed for each genotype. According to the manufacturer’s instruction, we used the AxyPrep™ Multisource Total RNA Miniprep Kit (Axygen) to extract total RNA, which was then retro-transcribed by using PrimeScript™ RT Reagent Kit with gDNA Eraser (Takara). Quality and concentration of the RNA extracted was checked with electrophoresis on 1% agarose gel and measured using the Nanodrop ND-2000 spectrophotometer (NanoDrop Technologies, USA). Concentration of the RNA samples used for cDNA synthesis was normalized by dilution with RNase-free ultra pure water.

qRT-PCR assays of 20 μL reaction volumes, which contained 0.5 μL of synthesized cDNA, 0.4 μM of gene-specific primers and 10 μL of SYBR® Premix Ex Taq™ (TaKaRa), were conducted by using ABI 7500 Real-time PCR System (Applied Biosystems). Following the manufacturer’s instruction, the qRT-PCR conditions were set up as: denaturing at 95 °C for 30 s, then 40 cycles of 95 °C for 5 s, 55 °C for 30 s and 72 °C for 30 s. To standardize the quantification of gene expression, we used the rice *Ubiquitin* (*UBQ*) gene (Os03g0234200, http://rapdb.dna.affrc.go.jp/) as an internal control. The data were analyzed according to the relative quantification method [26]. Primers for qRT-PCR of *UBQ*, *OsMADS51*, *Ehd1*, *Hd3a*, *RFT1*, *Hd1* and *OsSPL2* were listed in Additional file 4: Table S2.

### RNA-Seq analysis

Two homozygous genotypes derived from CJ1 were grown in controlled chambers treated with high and low temperature under long day (LD) conditions, which were set as 34 °C, 14-h light / 28 °C, 10-h dark and 26 °C, 14-h light/20 °C, 10-h dark, respectively. Penultimate leaves of the two genotypes were collected in 23 days after sowing (DAS) at high temperature and 44 DAS at low temperature with two biological replicates. In this condition, heading date of the early-heading genotype was 49 d at high temperature and 70 d at low temperature. Based on the understanding that the duration from panicle initiation to heading in early-season *indica* rice is constantly about 28 d, the 23 DAS for high temperature and 44 DAS at low temperature both correspond to about 26 d before heading, covering the transition period from vegetative to reproductive phase. Total RNA was extracted using the TRIzol reagent (Invitrogen), according to the manufacturer’s protocol. RNA quantification and qualification, library preparation for transcriptome sequencing, clustering and sequencing, data analysis and quality control were conducted by Biomarker (http://www.biomarker.com.cn/) according to their protocol.

Differential expression analysis between the two genotypic groups was performed using the DESeq R package (1.10.1) with a model based on the negative binomial distribution. The resulting *P* values were adjusted for controlling the false discovery rate, using the Benjamini and Hochberg’s approach. Genes with an adjusted *P*-value < 0.01 and log2 fold change above or below 1.0 were designated as differentially expressed genes (DEGs). For validation of critical genes identified from the RNA-seq analysis, qRT-PCR was performed by using the same samples for the RNA-seq.

### Statistical analysis

For the replicated field trials, phenotypic differences between the two different genotypic groups in each NIL set were evaluated by two-way analysis of variance (ANOVA), which was performed with SAS procedure GLM (SAS Institute Inc. 1999) as described previously [27]. When a significant difference (*P* < 0.05) was found between the two genotypes, the QTL effects, including additive effect and the proportion of phenotypic variance explained, were estimated with the same data and model.

For NIL set that was grown in the controlled chamber, one-way ANOVA was conducted to test the phenotypic differences between the two homozygous genotypic groups.

## Results

### Effect of *qHd1* on the tolerance to high temperatures shown in the multiple sowing-date experiment

#### Photoperiod and temperature conditions

With the sowing date changing at a specific rice cropping area, the available photo-temperature resources and the development progress of a rice variety varied greatly. Five different sowing dates were applied in this study, lasting from early to late cropping season in 2015 in Hangzhou (Table 1).

Day length and temperature are two key factors of environmental conditions and closely associated with seasonal changes. As for day length, 13.0 h could be considered as the threshold distinguishing SD from LD conditions for rice [22–24]. In the multiple-sowing experiment, the day length increased from 13.3 h at 28 April to 14.1 h at 23 June, and then decreased to 13.0 h at 24 August (Additional file 5: Figure S3). Generally, the duration from panicle initiation to heading in rice stably takes about 28 d [5, 28–30]; that is to say, rice plants flowering before 20 September could experience the transition from vegetative to reproductive phase under LD conditions. Given that, we purposed that all the lines tested in this experiment have completed the phase transition under LD conditions. As for daily mean temperature, a warming trend from early to late cropping seasons was found, despite of obvious fluctuations (Additional file 5: Figure S3). The average temperatures from sowing to heading were 23.6 °C in Trial I, 24.1 °C in Trial II, 24.9 °C in Trial III, 27.1 °C in Trial IV, and 27.7 °C in Trial V. From these observations, we inferred that temperature would be a more critical environmental variable than day length in this test.

#### QTL effects on heading date

**Fig. 1:**
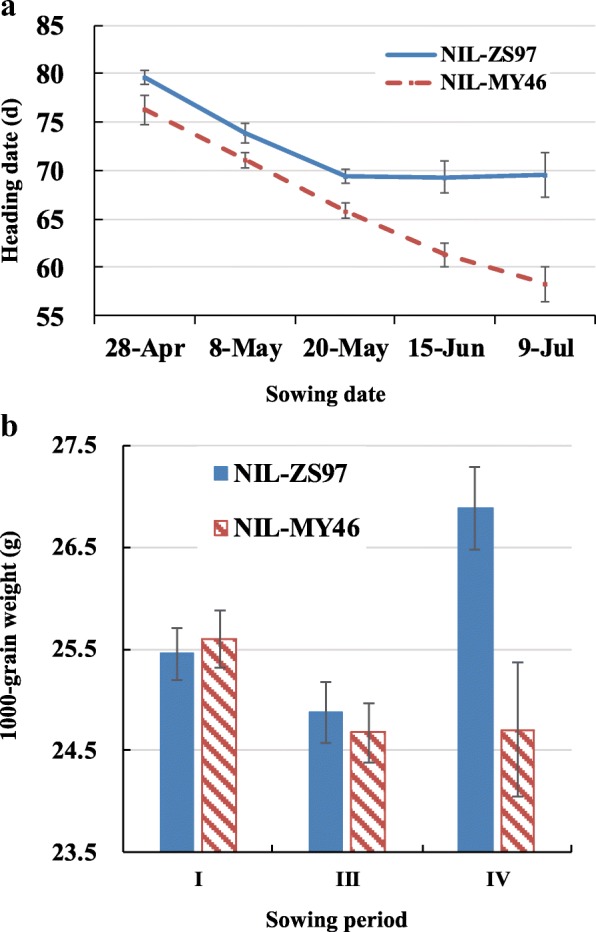
Effects of *qHd1* on heading date (**a**) and 1000-grain weight (**b**) detected in the multiple sowing-date experiment. I, III and IV in (**b**) refer to the trials with sowing date of 28 Apr, 20 May, and 15 Jun in (**a**), respectively. In all the trials, 50 lines of each genotype in the CJ1 and CJ2 populations were tested. Values are represented as means ±SD. NIL-ZS97 and NIL-MY46 are near isogenic lines with Zhenshan 97 and Milyang 46 homozygous genotypes at *qHd1*, respectively

In all the five trials, the ZS97 homozygous lines always had a late heading as compared to the MY46 homozygous lines. With the postponement of sowing date from Trial I to V, HD of MY46 homozygotes continued to decrease for about 5 d per trial from 76 to 58 d, while that of ZS97 homozygotes was promoted at the same rate in the first three trials but then stabilized at 69 d (Fig. 1a).

Two-way ANOVA was conducted to test the heading-date difference between the two genotypic groups of each NIL population. As shown in Table 2, highly significant genotypic effects (*P* < 0.0001) were detected in both populations in each trial, explaining 54.0–90.4% of the phenotypic variances. Notably, the additive effects of *qHd1* were similar among the first three trials and then increased in the last two ones: the averaged effects of ZS97 allele on delaying heading over CJ1 and CJ2 were ranged from 1.4 to 1.8 d in Trial I, II and III, while the effects became 4.0 and 5.7 d in Trial IV and V, respectively. These results support the notion that *qHd1* mediated a floral response to temperature variation with the allele from ZS97 conferring a stable heading date at high temperatures.

**Table 2:** Effects of *qHd1* on heading date tested in the multiple sowing-date experiment

Trial	Population	Phenotypic mean^a^		*P*	*A* ^b^	*R*^2^(%)^c^
(Sowing date)	name	NIL^ZS97^	NIL^MY46^			
Trial-I (28 Apr)	CJ1	79.3	76.0	< 0.0001	−1.6	58.6
	CJ2	79.9	76.5	< 0.0001	−1.7	59.4
Trial-II (8 May)	CJ1	74.1	71.3	< 0.0001	−1.4	54.0
	CJ2	73.7	70.9	< 0.0001	−1.4	62.0
Trial-III (20 May)	CJ1	69.3	66.1	< 0.0001	−1.6	76.8
	CJ2	69.5	65.7	< 0.0001	−1.9	81.2
Trial-IV (15 Jun)	CJ1	69.1	61.5	< 0.0001	−3.8	79.6
	CJ2	69.5	61.2	< 0.0001	−4.2	90.4
Trial-V (9 Jul)	CJ1	69.6	58.3	< 0.0001	−5.6	81.6
	CJ2	69.6	58.2	< 0.0001	−5.7	88.4

^a^NIL^ZS97^ and NIL^MY46^ are near isogenic lines having homozygous *qHd1* alleles from Zhenshan 97 and Milyang 46, respectively

^b^additive effect of replacing a Zhenshan 97 allele with a Milyang 46 allele

^c^proportion of phenotypic variance explained by the QTL effect

#### QTL effects on yield traits

According to the HD distribution curves of ZS97 homozygotes across the five treatments (Fig. 1a), we easily identified the declining trend from Trial I to III and the steady trend from Trial III to V with the turning point in Trial III. Therefore, Trial I, III and IV were selected as the representatives to further analyze the effects of *qHd1* on yield traits.

Two-way ANOVA was performed to test phenotypic differences of yield traits between the two homozygous genotypic groups of each NIL population, and the results are presented in Table 3. When a significant effect (*P* < 0.05) was detected, the enhancing alleles were always derived from ZS97 except for the small effects detected on TGW in CJ1 in Trial I and on SF in CJ2 in Trial III. This is in accordance with the understanding that late-maturing allele is generally associated with improving performance of yield traits.

**Table 3:** Effects of *qHd1* on yield traits in Trail-I, III and IV of the multiple sowing-date experiment

Trial	Trait	CJ1-Phenotypic mean^a^		*P*	*A* ^b^	*R*^2^ (%)^c^	CJ2-Phenotypic mean		*P*	*A*	*R*^2^ (%)
(Sowing date)		NIL^ZS97^	NIL^MY46^				NIL^ZS97^	NIL^MY46^			
I (28 Apr)	NP	6.61	6.43	0.1769			6.82	6.46	0.0087	−0.18	3.6
	NGP	109.4	109.4	0.9649			105.2	106.7	0.3825		
	TGW	25.50	25.71	< 0.0001	0.11	9.5	25.40	25.49	0.1194		
	SF	88.17	89.02	0.1391			88.13	88.15	0.9611		
	GY	17.36	16.70	0.0599			17.56	16.53	0.0086	−0.51	3.6
III (20 May)	NP	7.16	7.15	0.9210			6.65	6.71	0.6642		
	NGP	110.1	105.6	0.0001	−2.28	7.3	115.0	108.9	< 0.0001	−3.07	9.9
	TGW	24.74	24.56	0.0002	−0.09	8.1	25.02	24.80	0.0004	−0.11	8.4
	SF	83.23	83.46	0.5555			83.94	84.88	0.0418	0.47	2.0
	GY	18.55	17.72	0.0500			18.32	17.57	0.0701		
IV (15 Jun)	NP	8.46	8.59	0.5240			7.82	7.83	0.9624		
	NGP	101.4	98.9	0.1892			106.5	105.6	0.5625		
	TGW	27.04	25.06	< 0.0001	−0.99	68.3	26.73	24.36	< 0.0001	−1.19	79.6
	SF	82.11	84.20	0.0005	1.05	7.3	83.34	85.32	0.0014	0.99	5.9
	GY	20.74	18.96	0.0006	−0.89	6.6	20.57	18.37	< 0.0001	−1.10	9.8

*NP* number of panicle per plant, *NGP* number of grains per panicle, *TGW* 1000-grain weight (g), *SF* spikelet fertility rate (%), *GY* grain yield per plant (g)

^a^NIL^ZS97^ and NIL^MY46^ are near isogenic lines having homozygous *qHd1* alleles from Zhenshan 97 and Milyang 46, respectively

^b^additive effect of replacing a Zhenshan 97 allele with a Milyang 46 allele

^c^proportion of phenotypic variance explained by the QTL effect

In Trial I, significant genotypic effects were detected on NP, TGW and GY, but none of them were consistent across the two NIL populations. In Trial III, yield component traits NGP and TGW, but not NP, showed significant variation in both populations, while the significance of GY variation was just marginal (*P =* 0.0500 in CJ1 and *P =* 0.0701 in CJ2). In Trial IV, highly significant and relatively large variation was observed on TGW and GY in both populations, while a small variation with opposite allelic direction was found on SF. Notably, TGW of ZS97 homozygotes were much larger than that of MY46 ones, with the additive effects and *R*^2^ averaged as 1.09 g and 74.0% over the two populations, respectively, which were the main sources of variation for GY.

Variation of TGW across the three trials was especially noteworthy when it was compared with variation of temperature and HD. As compared to MY46 homozygotes, ZS97 homozygotes greatly increased TGW in Trial IV but not in Trial I and III (Fig. 1b). The average increase over the two populations was 8.8% in Trial IV, whereas differences between the two genotypes were all less than 1% in Trial I and III (Table 3). When considering the increase of temperature with postponed sowing date (Additional file 5: Figure S3), it is reasonable to infer that the *qHd1*^ZS97^ allele is favorable for achieving high grain weight at high temperature. When further considering the stable HD of ZS97 homozygotes under high temperature, it is evident that the *qHd1*^ZS97^ allele would be advantageous for realizing a higher grain yield through its tolerance to heading date acceleration at high temperatures.

### Validation of the floral response of *qHd1* to temperature by the temperature-gradient test in phytotron

**Fig. 2:**
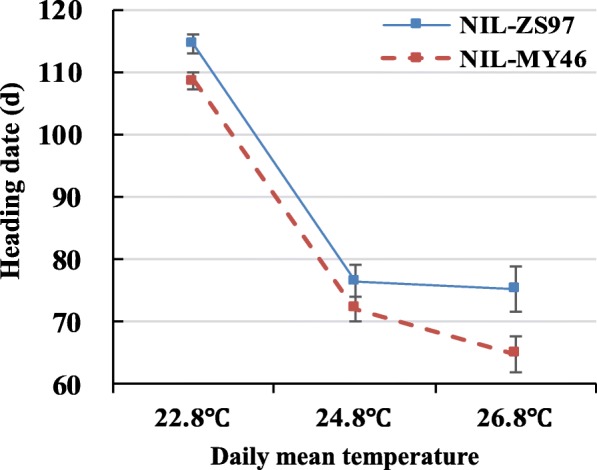
The temperature-gradient test in phytotron. Heading date of NIL-ZS97 and NIL-MY46 were the averaged data of 16 plants for each genotype. Values are represented as means ±SD

NIL sets which included two homozygous genotypes derived from CJ1 differing at *qHd1* was examined in the controlled chambers with increasing ambient temperature under SD conditions. As shown in Fig. 2, with the daily mean temperature increase from 22.8 to 24.8 °C, sharp decrease of heading date was observed for both genotypes, i.e., from 114.6 to 76.6 d for ZS97 homozygotes and from 108.7 to 72.2 d for MY46 homozygotes. But when further increase to 26.8 °C, stabilization of HD in ZS97 homozygotes were observed with minor decrease of just 1.4 d, in contrary to the great decrease of 7.3 d in the MY46 homozygotes. Across all the three temperature treatments, highly significant difference in HD (*P* < 0.0001) was found between the two genotypic groups. These results further indicate the involvement of *qHd1* in stabilizing rice heading at high temperatures and possible existence of a thermosensory pathway it mediated. In addition, consistent performance of *qHd1* under the SD condition in the phytotron and NLD condition in the paddy field suggests that this thermosensory pathway is independent of day length.

### The floral pathway underlying *qHd1-*mediated thermosensory response

**Fig. 3:**
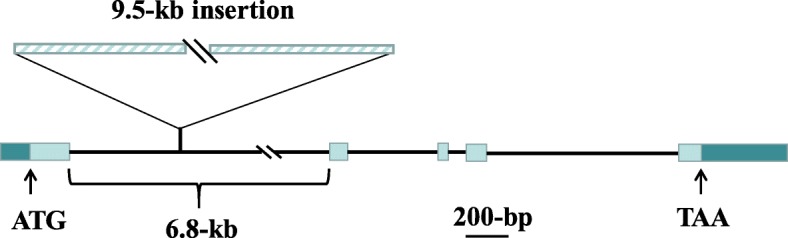
A large sequence structure variation of 9.5-kb in the 1st intron of *OsMADS51*. Zhenshan 97 contains a 9.5-kb insertion as compared to Milyang 46

The target region contains ten annotated genes (http://rapdb.dna.affrc.go.jp/), including two genes having function related to heading date, i.e., *OsMADS51* [31] and *OsSPL2* [32]. We sequenced the promoter and entire gene regions of *OsMADS51* and *OsSPL2* from the two parental lines, ZS97 and MY46. Sequence comparison between ZS97 and MY46 identified no polymorphism in the promoter and exon regions. At the same time, a large insertion of 9.5-kb was detected in the 1st intron of *OsMADS51* in ZS97, as compared to MY46 (Fig. 3 and Additional file 6: Figure S4). This result prompted us to examine whether this large structure variation was a functional polymorphism that has an influence on gene expression. Using qRT-PCR, we tested *OsMADS51*, and four key heading-date regulators, i.e., florigen genes *Hd3a* and *RFT1* and their upstream signal integrators *Hd1* and *Ehd1* [11, 12]. *OsSPL2* was also included, because it is located in the *qHd1* region and annotated to be functionally related to heading.

The effect of *qHd1* on HD became much larger in Trial IV than the three previous trials (Fig. 1a), indicative of the influence of temperature in heading. Therefore Trial IV was chosen for gene expression analysis. Average HD in this trial was 69.3 d for ZS97 homozygotes and 61.4 d for MY46 ones. Gene expression was assessed in 30, 35 and 45-day-old plants grown in the field under NLD conditions, well covering the transition period from vegetative to reproductive phase.

**Fig. 4:**
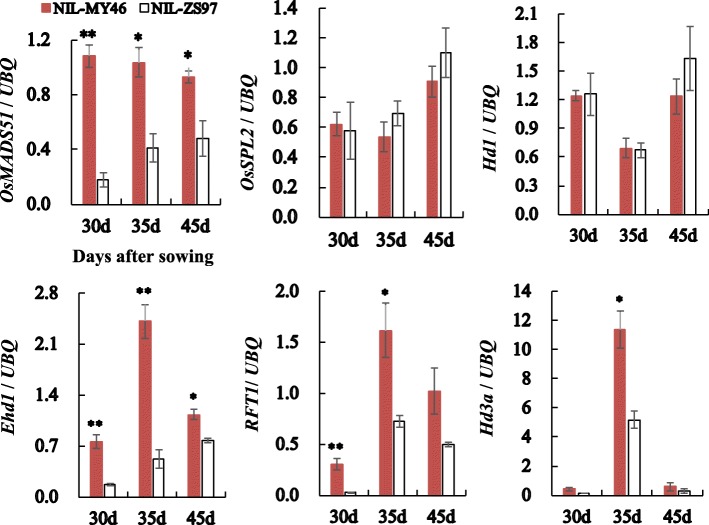
Comparisons of the gene expression. *OsMADS51*, *OsSPL2*, *Hd1*, *Ehd1*, *RFT1* and *Hd3a* were tested between ZS97 and MY46 homozygous lines of the CJ1 population by qRT-PCR in 30, 35, and 45 days after sowing. Values are represented as means ± SE, derived from three biological replicates with two technical repetitions each. *UBQ*, ubiqutin used to normalize the values. *P < 0.05 or **P < 0.01, by Student’s t-test

As illustrated in Fig. 4, significant changes in the transcript levels between the two genotypic groups were found for the most potential candidate, *OsMADS51*, and key heading-date regulators *Ehd1*, *RFT1* and *Hd3a*. Lower expression was found in NIL-ZS97 than NIL-MY46, which is in accordance with late heading in NIL-ZS97. The largest fold changes were found in the first or second sampling time, including 6.2 fold of *OsMADS51* in 30 DAS, 4.6 fold of *Ehd1* in 35 DAS, 11.3 fold of *RFT1* in 30 DAS, and 3.7 fold of *Hd3a* in 30 DAS. These results suggest that the 9.5-kb sequence structure variation in *OsMADS51* could underlie a *qHd1*-mediated heading response to temperature, by regulating the *Ehd1-RFT1/Hd3a* expression.

### Distinct expression regulation of *qHd1* between high and low temperatures revealed by RNA-seq

In the phytotron experiment under LD conditions, heading date of the NIL-MY46 and NIL-ZS97 were 48.5 and 64.9 d under high daily mean temperature of 31.5 °C, and 70.3 and 78.3 d under low daily mean temperature of 23.5 °C, respectively. The 16.4 d difference between the two NILs at high temperature was much larger than the value of 8.0 d at low temperature, showing the response of *qHd1* to temperature variation. It is also noted that the underlying gene *OsMADS51* encoded an MADS-box transcription factor, regulating the expression of genes that are related to metabolism and signaling in response to environmental variations [33, 34]. Therefore, RNA-seq was performed to identify downstream genes involved in *qHd1*-mediated thermosensory network.

**Fig. 5:**
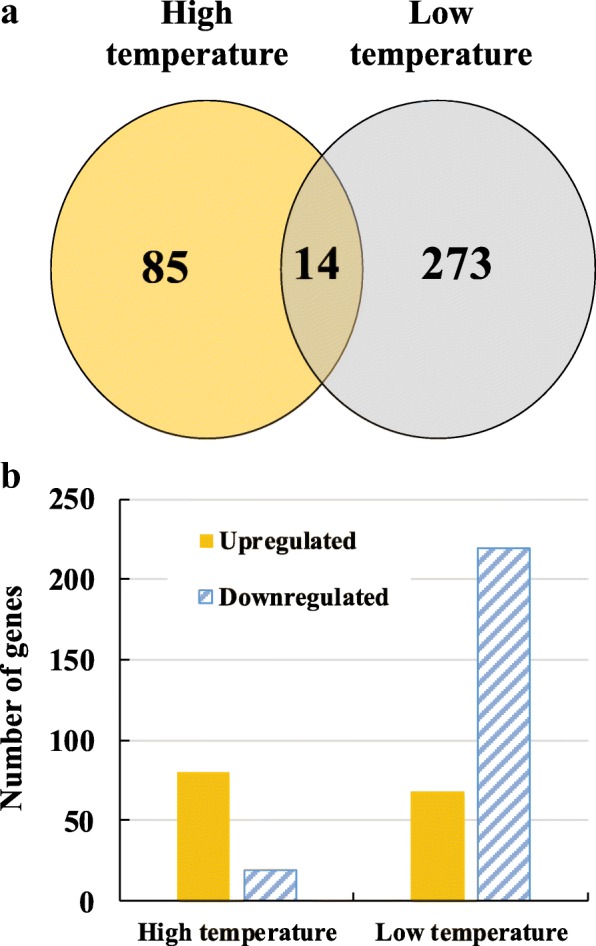
Overview of differentially expressed genes identified by RNA-seq. **a** Venn diagram illustrates unique and common genes differentially regulated in the high- and low-temperature treatment. **b** Histogram shows the number of genes up-regulated and down-regulated in the high- and low-temperature treatments of NIL-ZS97 as compared to NIL-MY46

Differentially expressed genes (DEGs) between NIL-ZS97 and NIL-MY46 were identified (Additional file 7: Table S3), showing large differences between the two treatments (Fig. 5). Ninety-nine DEGs were detected at high temperature, much fewer than 287 DEGs found at low temperature (Fig. 5a). As compared to NIL-MY46, the numbers of DEGs up- and down-regulated in NIL-ZS97 were 80 and 19 at high temperature, but 68 and 219 at low temperature (Fig. 5b). In addition, for the category of up-regulated genes at high-temperature treatment, seven of the top ten genes showing the largest expression change were found to participate in stress responses, including four genes related to cold conditions (Additional file 8: Table S4). For the remaining three category of DEGs, few of the top ten genes responded to adverse temperature stresses. These results implied that the expression-regulation pattern of *qHd1* may be differentiated in response to temperature variation, and further confirm the involvement of *qHd1* in thermosensory pathway.

Of the DEGs identified, only 14 were commonly found in the two treatments. While *OsMADS51*, *Ehd1* and *RFT1* were detected as common DEGs, no other heading-date genes exhibited significant differential expression. Considering that high homology and close linkage between *RFT1* and *Hd3a* may prevent the separate detection of them, *Hd3a* was subject to qRT-PCR analysis using RNA samples applied for RNA-seq, together with *OsMADS51*, *Ehd1* and *RFT1*. All the four genes showed significant expression differences as expected (Additional file 9: Figure S5), suggesting a new pathway *OsMADS51*-*Ehd1*-*RFT1*/*Hd3a* for the regulation of flowering in response to temperature.

### Differentiation of *qHd1* between early-season and middle/late-season *indica* rice varieties

As described above, the *qHd1* allele derived from the early-season *indica* rice variety ZS97 conferred tolerance to high temperature which is a common environmental stress encountered during the heading and grain-filling period of early-season rice cropping. We therefore decided to test whether the beneficial allele with the 9.5-kb insertion has been commonly utilized in rice production. One-hundred and nine *indica* rice varieties widely used in China were collected and analyzed (Additional file 3: Table S1).

Eleven pairs of PCR primers were used for genotyping of *qHd1*. Ten pairs extending from 1F/1R to 10F/10R jointly spanned the entire 9.5-kb insertion of the ZS97 allele (Fig. 3 and Additional file 6: Figure S4). Meanwhile, primer pair 1F/10R amplified a 1.0–kb fragment from MY46. Among the 13 early-season *indica* rice varieties that had large planting areas for a long period before 1985, all except one carried the 9.5-kb insertion. Among the 82 major *indica* rice varieties used during 1990−2014, the 9.5-kb insertion was identified in 43 of the 48 early-season varieties, but only detected in three of the 24 middle-season and two of the ten late-season varieties. For the 14 early-season *indica* rice varieties released after 2000, including the backbone parents Jiayu 253 and Zhongxuan 181, and 12 descendants of them, the 9.5-kb insertion was found without exception. These findings indicate that *qHd1*/*OsMADS51* could have undergone intensive artificial selection and play a significant role in the adaptation of early-season *indica* rice to high temperature at the heading and grain-filling stages.

## Discussion

High temperature commonly has negative impacts on rice development and causes yield losses. Rice heading is generally accelerated when temperature increased. The shortened vegetative-growth duration accompanied by limited source build-up is often a severe constraint for attaining a high yield [35]. During the reproductive growth and grain-filling stages, elevated temperature could result in reduced starch accumulation in developing grain because of the declined starch biosynthesis and earlier cessation of assimilate translocation [36]. Identification of genes associated with tolerance to heading date acceleration is of great importance for rice breeding. In the present study, we identified the thermosensory features of *qHd1*, a minor QTL for heading date that was preciously fine-mapped [19]. Results showed that the allele derived from the early-season *indica* rice variety ZS97 conferred stable heading date with higher grain weight and grain yield under high temperatures. It was also found that this allele is predominantly shared by early-season *indica* rice varieties, but few middle- and late-season rice varieties carry this allele. Considering the frequent occurrence of high temperature stress in both the early- and middle-season rice cropping especially in recent years, introgression of the beneficial allele at *qHd1* from early-season rice to middle-season rice would be greatly helpful for overcoming yield losses due to heat stresses.

Another finding of this study is the *OsMADS51-Ehd1-RFT1/Hd3a* pathway underlying the *qHd1*-mediated floral response to temperature. The *Ehd1-RFT1/Hd3a* pathway downstream of *OsMADS51* also functioned as integrators of drought signals [37]. The convergence of heat and drought signal on the heading-date pathway implied the regulatory connection between stress and flowering. As it is well-known, plants generally perceive and integrate various environmental signals to determine the proper timing of phase transition for maximizing reproductive success, either rapidly induce flowering before the stress becomes a great detrimental or temporarily inhibit heading until the stress is over [38]. Consistent with this view, *Grain number, plant height and heading date 7* (*Ghd7*), the major floral repressors upstream of *Ehd1* and the two florigen genes in rice [39], was reported to respond to multiple abiotic stress, including drought, abscisic acid, jasmonic acid, high temperature and low temperature [40]. Likewise, in our results of transcriptome profiling, the *OsMADS51/qHd1-*mediated expression change was observed for a group of genes that were involved in multiple abiotic and biotic stress-response pathways such as cold, heat, drought, salinity, disease and insect (Additional file 8: Table S4). These results suggest that the heading-date genes/pathway might play a key role in integrating the floral transition and stress response, which would provide new leads for exploring the intricate genetic networks.

Day length and temperature are two key environmental factors associated with seasonal changes and strongly affect the crop duration and grain yield of rice. Recent studies have revealed that heading-date genes could possessed pleiotropism for yield traits, and their natural variations have been used in rice breeding mainly with the late-maturing allele enhancing grain yield, such as *Ghd7* [39], *Days to heading 8/Grain number, plant height and heading date 8* [41, 42], *Hd1* [43], *Grain number, plant height and heading date 7.1 /Days to heading 7* [44, 45], and *RFT1* [46]. These reports focus on the pleiotropism for yield traits in response to photoperiodic changes, and none of them work on the response to temperature variation. In the multiple sowing-date experiment of this study, we noted the pleiotropism of *OsMADS51/qHd1* varied greatly in response to temperature change (Table 3). Variation in grain yield might be derived from both NGP and TGW (Trial III) or mainly from TGW (Trial IV). The magnitude of genotypic effects on yield traits also varied greatly, showing an association of stronger effects with increased daily-mean temperature (Additional file 5: Figure S3). This finding was consistent with the reports that a slight temperature change of 1–2 °C would have dramatic effects on grain yield and its components [2, 3, 47].

*OsMADS51*, the gene underlying *qHd1*, was previously isolated by T-DNA insertion and reported to be photoperiod sensitive, influencing heading date under SD conditions only [31]. However, this gene also showed contribution to heading-date variation under LD conditions in this study. These suggest that *OsMADS51* could affect heading under both under the SD and LD conditions, but the function may change depending on the genetic backgrounds. For the temperature responsive function of *OsMADS51*, no similar result has been reported for heading date genes in rice. Nevertheless, it is noted that *OsMADS51* encodes a MADS-domain transcription factor, and several key components such as *SHORT VEGETATIVE PHASE* [48], *FLOWERING LOCUS M* [49], *FLOWERING LOCUS C* and *MADS AFFECTING FLOWERING 2, 3, 4 and 5* [50] in the *Arabidopsis* flowering-regulatory thermosensory pathway also belong to the MADS-box transcription factor family. This is in support of the inference that *OsMADS51* mediates the floral response to temperature variation in rice.

## Conclusions

Our work identified the genetic contribution of *qHd1* to temperature responses in rice, showing that this QTL regulates heading date in association with high-temperature tolerance at the heading and grain-filling stages. The underlying floral pathway *OsMADS51-Ehd1-Hd3a/RFT1* was found, in which *OsMADS51* is the gene for QTL *qHd1*. The beneficial allele of *OsMADS51* has become predominant in early-season *indica* rice varieties but remains rare in the middle- and late-season ones. This indicates the great importance of *qHd1* for the adaptation of early-season *indica* rice to high temperature during the yield-forming process. The information provides new leads for a molecular understanding of how temperature influences rice growth and then affects rice production.

## Additional files

Additional file 1: **Figure S1.** Frequency distribution of heading date in the two NIL populations tested in Lingshui in Dec 2013–Apr 2014. NIL-ZS97 and NIL-MY46 are near isogenic lines with Zhenshan 97 and Milyang 46 homozygous genotypes at *qHd1*, respectively. (PPT 119 kb)

Additional file 2: **Figure S2.** Development of two sets of near isogenic lines segregated for *qHd1* and genotypic compositions of the two populations in the target region. (PPT 118 kb)

Additional file 3: **Table S1.** Three collections of Chinese *indica* rice varieties tested in this study. (XLSX 17 kb)

Additional file 4: **Table S2.** Sequences of the primers used for quantitative real-time PCR. (DOCX 13 kb)

Additional file 5: **Figure S3.** Daily mean temperature and day length variation observed in the multiple-sowing experiment conducted in Hangzhou in 2015. The data of daily mean temperature were derived from the weather station located near the experimental field, while day length data were from www.timeanddate.com. I, II, III, IV and V refer to different trials with sowing date of 28 Apr, 8 May, 20 May, 15 Jun and 9 Jul, respectively. Gray columns represent the duration from sowing to heading in each trial, with the corresponding averaged temperature. (PPT 132 kb)

Additional file 6: **Figure S4.** Genomic sequence of *OsMADS51* in Zhenshan 97, showing the 9.5-kb insertion as compared to Milyang 46. The sequence of 3′ and 5’-UTR is shaded in green, exons in yellow, and the 9.5-kb insertion in grey. Ten pairs of primers used for sequencing and genotyping of the 9.5-kb insertion is indicated by colored and underlined letters, with forward primers in red and reverse primers in golden, e.g., 1F/1R – 10F/10R. (PDF 449 kb)

Additional file 7: **Table S3.** Differentially expressed genes detected by RNA-seq in high- and low-temperature treatment, classified by regulation direction and ranked by log2FC. (XLSX 118 kb)

Additional file 8: **Table S4.** Differentially expressed genes responding to adverse stress. (XLSX 16 kb)

Additional file 9: **Figure S5.** Validation of RNA-seq profile in the high-temperature (a) and low-temperature (b) treatment by expression analysis of *OsMADS51*, *Ehd1*, *RFT* and *Hd3a* using qRT-PCR. Values are represented as means ± SE, derived from two biological replicates with two technical repetitions each. *UBQ*, ubiqutin used to normalize the values. (PPT 116 kb)
