# Supplementary figures and images for: Natural variation at qHd1 affects heading date acceleration at high temperatures with pleiotropism for yield traits in rice
Source: BMC Plant Biol. 2018 Jun 7;18:112. doi: 10.1186/s12870-018-1330-5 (PMC5992824; doi:10.1186/s12870-018-1330-5)

## Slide 1
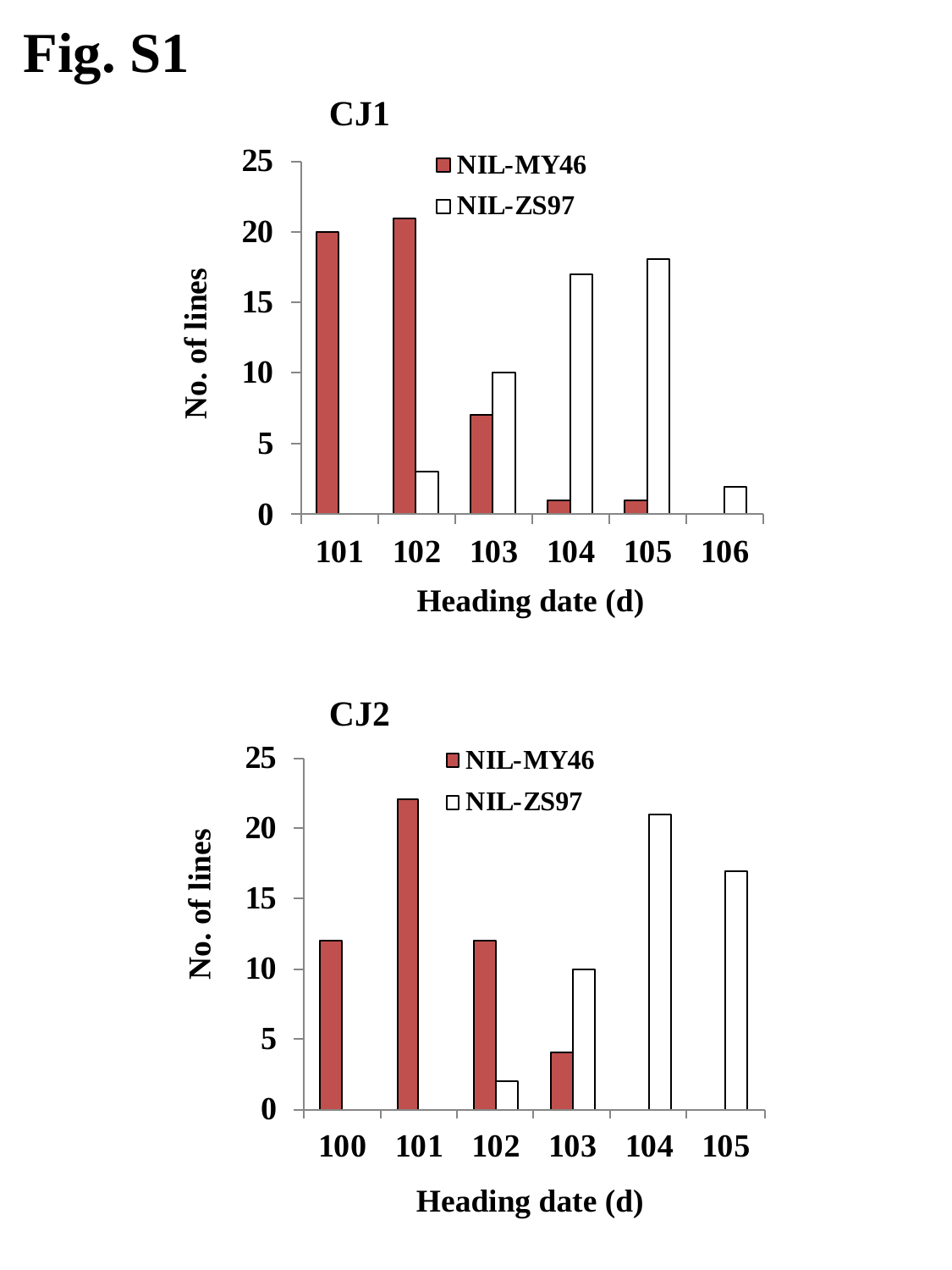

Fig. S1
CJ1
No. of lines
Heading date (d)
CJ2
No. of lines
Heading date (d)

Supplement: Supplementary file 1 — Figure S1. Frequency distribution of heading date in the two NIL populations tested in Lingshui in Dec 2013–Apr 2014. NIL-ZS97 and NIL-MY46 are near isogenic lines with Zhenshan 97 and Milyang 46 homozygous genotypes at qHd1, respectively. (PPT 119 kb) [file 12870_2018_1330_MOESM1_ESM.ppt]

## Slide 1
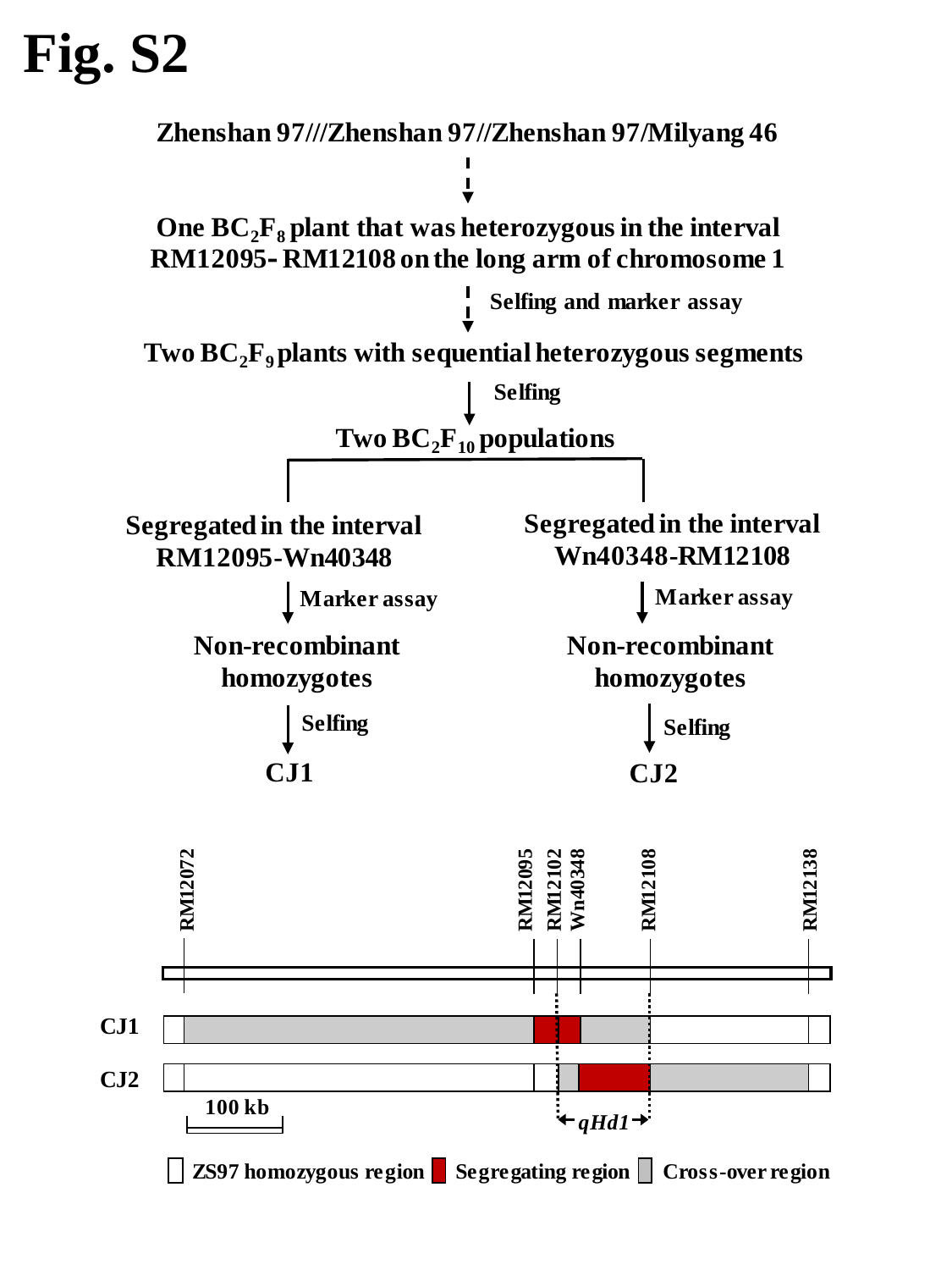

Fig. S2

Supplement: Supplementary file 2 — Figure S2. Development of two sets of near isogenic lines segregated for qHd1 and genotypic compositions of the two populations in the target region. (PPT 118 kb) [file 12870_2018_1330_MOESM2_ESM.ppt]

## Slide 1
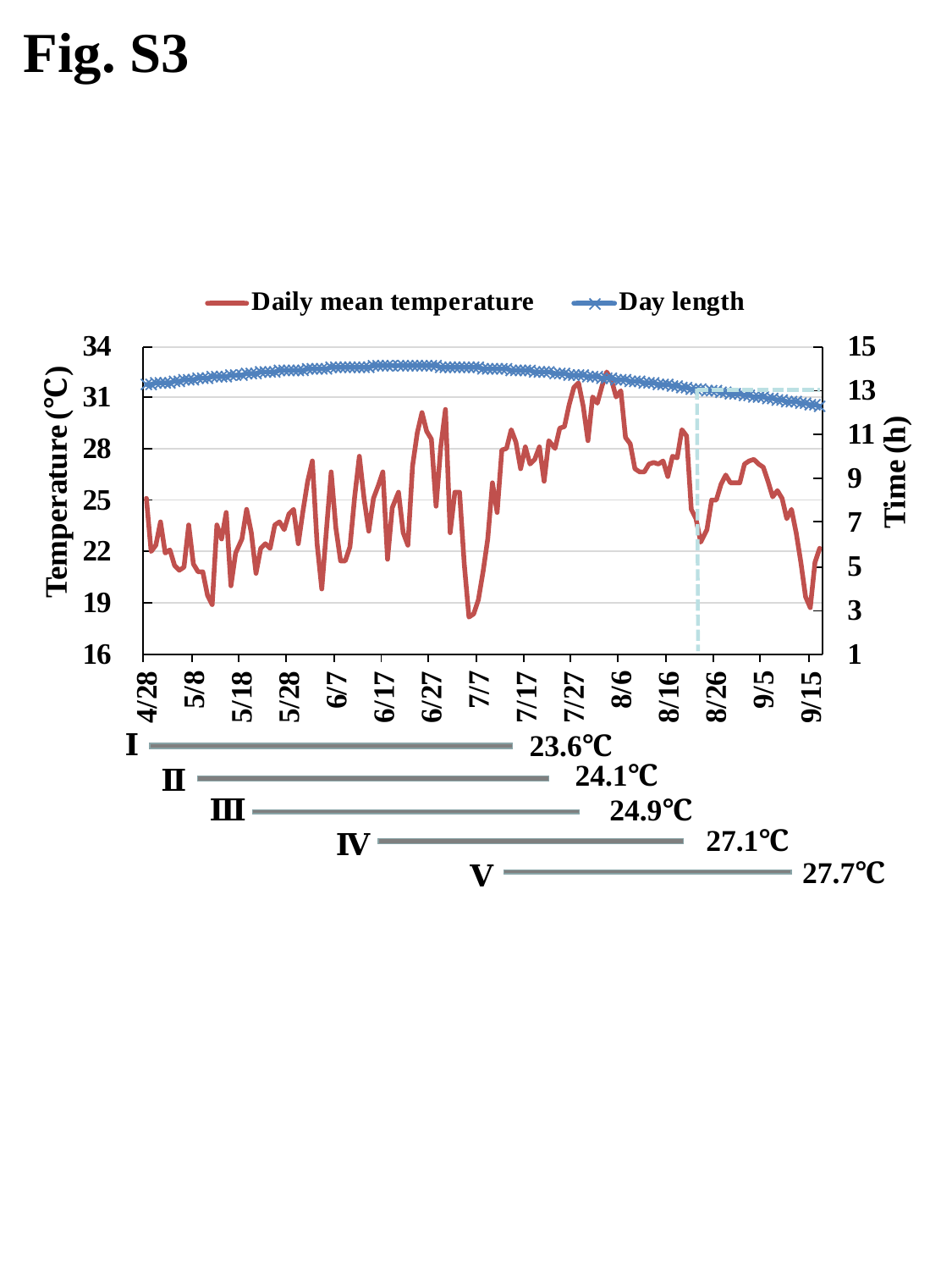

Fig. S3

Supplement: Supplementary file 5 — Figure S3. Daily mean temperature and day length variation observed in the multiple-sowing experiment conducted in Hangzhou in 2015. The data of daily mean temperature were derived from the weather station located near the experimental field, while day length data were from www.timeanddate.com. I, II, III, IV and V refer to different trials with sowing date of 28 Apr, 8 May, 20 May, 15 Jun and 9 Jul, respectively. Gray columns represent the duration from sowing to heading in each trial, with the corresponding averaged temperature. (PPT 132 kb) [file 12870_2018_1330_MOESM5_ESM.ppt]

## Slide 1
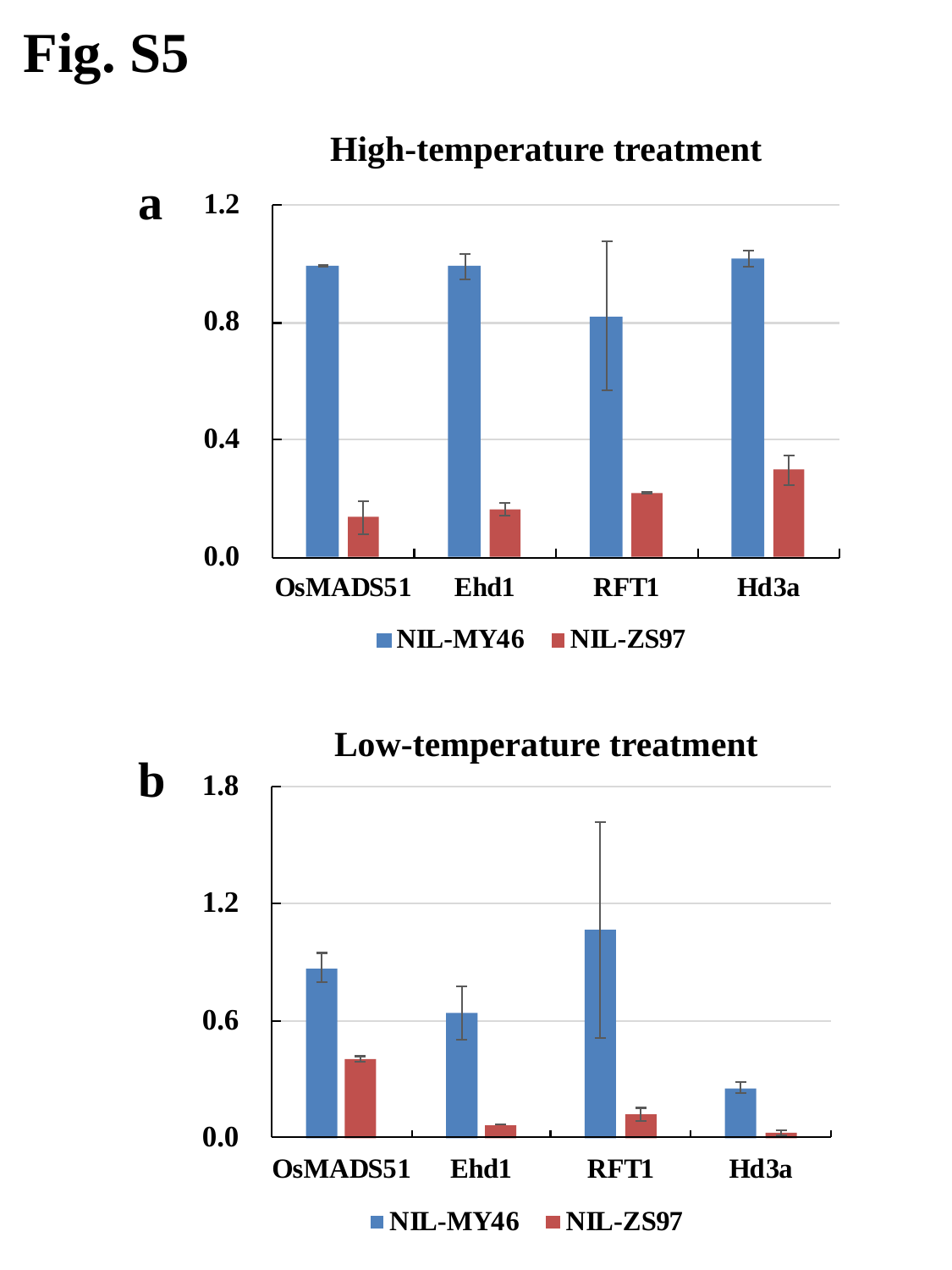

Fig. S5
High-temperature treatment
a
Low-temperature treatment
b

Supplement: Supplementary file 9 — Figure S5. Validation of RNA-seq profile in the high-temperature (a) and low-temperature (b) treatment by expression analysis of OsMADS51, Ehd1, RFT and Hd3a using qRT-PCR. Values are represented as means ± SE, derived from two biological replicates with two technical repetitions each. UBQ, ubiqutin used to normalize the values. (PPT 116 kb) [file 12870_2018_1330_MOESM9_ESM.ppt]
