# Supplementary material for: Natural variation at qHd1 affects heading date acceleration at high temperatures with pleiotropism for yield traits in rice
Source: BMC Plant Biol. 2018 Jun 7;18:112. doi: 10.1186/s12870-018-1330-5 (PMC5992824; doi:10.1186/s12870-018-1330-5)
Supplement: Supplementary file 4 — Table S2. Sequences of the primers used for quantitative real-time PCR. (DOCX 13 kb) [file 12870_2018_1330_MOESM4_ESM.docx]

**Table S2** Sequences of the primers used for quantitative real-time PCR.

| Primer name | Forward-primer sequence | Reverse-primer sequence |
| --- | --- | --- |
| *OsMADS51* | GAAATCAAAGAAGATGTTGGCAAA | CTTCCTCCTGCCCCCTAGAG |
| *OsSPL2* | CCGCAGCCTGATCCTCTCAACCC | GGCCTCCGTCTTGACCACGTT |
| *Hd1* | TCTCAGGTCCCTCGCTTCA | GCATACGCCTTTCTTGTTTCATA |
| *Ehd1* | CCTACAGTGATTATGGCTTCA | GTGCTGCCAAATGTTGCTC |
| *Hd3a* | GCTCACTATCATCATCCAGCATG | CCTTGCTCAGCTATTTAATTGCATAA |
| *RFT1* | TGACCTAGATTCAAAGTCTAATCCTT | TGCCGGCCATGTCAAATTAATAAC |
| *Ubq* | AACCAGCTGAGGCCCAAGA | ACGATTGATTTAACCAGTCCATGA |
