# Supplementary material for: Natural variation at qHd1 affects heading date acceleration at high temperatures with pleiotropism for yield traits in rice
Source: BMC Plant Biol. 2018 Jun 7;18:112. doi: 10.1186/s12870-018-1330-5 (PMC5992824; doi:10.1186/s12870-018-1330-5)
Supplement: Supplementary file 6 — Figure S4. Genomic sequence of OsMADS51 in Zhenshan 97, showing the 9.5-kb insertion as compared to Milyang 46. The sequence of 3′ and 5’-UTR is shaded in green, exons in yellow, and the 9.5-kb insertion in grey. Ten pairs of primers used for sequencing and genotyping of the 9.5-kb insertion is indicated by colored and underlined letters, with forward primers in red and reverse primers in golden, e.g., 1F/1R – 10F/10R. (PDF 449 kb) [file 12870_2018_1330_MOESM6_ESM.pdf]

Figure S4 Genomic sequence of OsMADS51 in Zhenshan 97, showing the 9.5-kb insertion as compared to Milyang 46. The sequence of 3' and 5'-UTR is shaded in green, exons in yellow, and the 9.5-kb insertion in grey. Ten pairs of primers used for sequencing and genotyping of the 9.5-kb insertion is indicated by colored and underlined letters, with forward primers in red and reverse primers in golden, e.g., 1F/1R – 10F/10R.

CGATCCCCTCCTCCTCCTCCCCCACTTCTCCCCACCACCACACCACCACACACACGCACGCACGCACGCACGC
CTCCGTTTTCCCCCACCGACGAGAGGCGGAGAGGGAGGGGAGCTAGGGTTTTTCGGGGCGGGGATGGCG
CGGAGGGGGAGAGTGCAGCTGAGGCGGATCGAGGACAAGGCGAGCCGGCAGGTGCGGTTCTCCAAGAG
GAGGGCGGGGCTGTTCAAGAAGGCGTTCGAGCTCGCCCTGCTCTGCGACGCGGAGGTGGCGCTCCTCGTC
TTCTCCCCCGCCGGCAAGCTCTACGAGTACTCCTCCTCCAGGTTCGCTCTCTGATTCCATCCCCCTCCCTCCCC
CCGCCTCCTCCTGTTGTTAGTTACGATCAAAACCCAGCCGCGGCCGAATCGAGACCCCCCGCCGCGGCAGG
ATCTGCCGATTGGTTCCGATCCCATTTCGTTCTGCTATCCCGGATCGATCGATGATTCTCCCTGCTATACTGTAT
CGTAATGGGTAAGCCTAGTGCACGCCATGTGCTCGAGACTATCTCGCAGAGGCCCCCCCCCACCCCCCCAA
CCCCCCACCGCCACCCACCGGCGTTGCGCTGCATGACAGCCCTGTGATTTCTGATTAGTAGGATCTTGTTCTC
TTAGCCTGTACAGTAGCAGCAGCCAGCAGCTTGTATCTAGTATATGAAGCTTTGACACCTTGTACTGTTCCGC
GCTTCGTCTCCCCGACGGCGTGGGCCGCGTTGTTTTTCCTCCCCTGGTGTGCTTTTACACGTGTTTCGCATGA
GGAGACACCAGCCTTCGTAGAGGCGGAGGCGCGCAGCAATACGCGCATCACCCTGAGAGCCACCGCAGTG
TACGGCCTACGGCGAGTGAGGAGTTTATCATTTTCTCCCAACTTTTTTTTCGGCATCTTGTTTTTTTTTTCTCT
GAGAGGGAGTGATGGAGGAGTACAGTTGTGTGTGTTTTCTTTAATTTATTTCAGCGACAGGGACGAAGTCG
GCTTGCTCTGTAGCGACCGGCCTATTCCCATAAATAGTCTTTTGTTATGGTATTGAGTGGCATATGTTAAATAAT
ATTATTTGATACAACTGCTTAAAGATTTTTTTGTAATGGCTTATTTCTTGTATTTTAGTGTACACATCGAACAGG
ACTATTCTTAGTGATATAGTAGTGGGATAACATATTTATCTAATTAACCTTTATTAATGATATACTATAAAGAACAT
CGATAATATATTTATATATGTCAAGCAACTTGATTGAAAAACAAGCACATCACTCACAAAGTTAGCCCAATTGT
GCACAATAGTCCATTTCATACCATGATGCTAAAAATCACGCGCTCTGATTGTTTGAGCGACAACCCAGAATAA
TTCATAGGTATAATATTTTTTATATATATTAAGTGGTTTAAAAACCAAGATTGAAAAATAGACCTCAATAAAAAA
ATGCCCCCTAAAATCAACTCTCAAGGAGAACCGCGTTTGGCTTTGGCCGATAATCCACGAACAAATGATGAA
ATCACATATTCCATATATTCGCATTCACTTATGAACAAATGGCAACTTTTCTTATTTCTTGGTAATGTACGCCCTA
CACATTTTCCAACCCACATTTTTCTAGTTTTGCACCCCCTCCACCACCAATATGGTACAACTCCAACTCTAAAA
TTTAGCTCCAGGAGTTAGGTCTGGAGTGGAGTTGCCTAAACCCAGTTCCACAACTcTAGTTTATTTTGTGAGA
GCTTCACCCAGCTTCACTCCCAATTTTAATGGAGCTGTAATTGTTTGGCTGAGCTCCAGCTCCAAGAGAAGT
GGAGCTAGAGCTGGAGCTGTGCCAAACAGGCCCTTAGCTTGTCCAAATGGTCTAGATTCTTATTTCAGATTC
TACGAGTCGGAAGGTGCATAATCAATAATCAATGAACTGGAAGCCAGAAGTTGGATAACGTAAGCTTCAGCT
AGATACTCACCAATTGCTTTTTAGGATCTGTAGCTTTCTTAAATAGGGCATTTCCTATCTTTAATACTCCATATGT
CCCAAAATATAACGACTTTTAGCCTTTAGAATTTGTTCCAAAATATTACAATTTCTCCACCAACATTCTCTTCTC
AACCAATCACAACCCTCCACCATTCAATTTCTCCACCTACATTCATTTCTCAACCAATCACAATCTTCCACCATT
TAATTCTACTTATTTTCTTAATAACCGTGACCAACTCTAAAACTCATTATATCCTGTCCGTCTCAAAATAAGTTTA
TTTTTCACGCATCTCACGCGTACCAATATCTAAGAAAAAAAAACTAGAATACCTTCACTTCATCAAATTCCAAT
GCAGTTGTTTCTCACTTTATCTACTCCTAATGTATTTTCCCCCTACTTTTACAAACTCTGTTGCAATAATTGCTAA
AATATGAACTTATTTTGGGATAAATGGGAGGGGCTAAAAATGATCTTATTCTGGGACGGATGGAGTAGATGC
TTCTTGTAGCATATGTTTTTGAATTTAACTACCATCAAGTTCCACCACAACATATAGAAGTATCTTGTAGTAACC
GTTATTTTGCTATTTTGCTAATGAAATAGACTAGAGGCATTTTAGCTTGCCTTGCACACGAGTGACAAGTAAA
CTAGAACGGAAGGAGAATTTTATTCCCCCTCAATCTGACCAAATCGATCCAAAGCCATACCAGTGTATGATTT
TCCTTACTAAATGTGAGGTTGATCTTTCACTAACAATGAATAAACTCTAAATTTTGAACTAGGATGGGACAATA

GACCCAAATTAACATAAAACTAGGAAATCAATGCGGCGATGTTTAAGTAATGACCATGATTGTAGACAACAA
AATAACACAAAATTGATAAAATAACTCGTACATGTATGTCATAGATTATTTAATATTTGTTGGGTCGTCCCCTTC
ATGTGTTAAATCATCTAGGAATTTGTCCTAACATTTGGGATAAAAAATTTCGCTAATAAATCTATTGAACATCTA
ATGTACGACAAAAGTCTCTATGACAACCCACTATTAGAAATTGGGGCATATAGATCTCCAGCAAACCTTAACA
TTTTATTCAAGGGTTTGTCCTTGAATACATCCAGGCAAAATGTTGAGGATTTCAATCAAAGTTCGGGCCATTG
GATTTATTTGACGCGTATCTTAATGGTTAACATGTGCCCTACCTCTTCTTACCTTTTCTCGTTATCTCACTATTGA
AGTTGTCCATGGTTACTAACACTCAAAGCCTAGAGGCTATACACGCACTACCTCCTGCTATTCCATATCACATT
CTAACCACAATGTCTACGGTTATTACTTATTAGCTATGTTTGCAATGCTTCGGGCTTGCATTCAACTAAGATAG
CAAAAAAAAAGTGTTAAAGTGTTAAAAAATCCCCACAATTCCCGGCACACGAGGGGGTGGGGGTTAAAAA
TCCCCTTGAATATAATTTGACTAGGTATCTCTTCTTTGTTGGCTTAGAAGATTTAGTTGTGTTTAGTACTTCATG
AAGTTATGGCTATCACTACAACTTGGTCAAGTTTGATTTCTTATCTAGTTGTTGCCATACTAATTAAAGTGTTGC
ATTTTGAAATGCACCCAAAGAAGTGAATTATGCACACATACTAGAATCATTAACAATCGTTTTTAAACTTGTTT
TATGAATACTAATATGTATTTTATACGCACGTAAGACACACATAATATGCTGACCATATATGTGATAAATTATGAA
TGATCTTATATCTTTTATACTCCCGTCGTTTAAAACTAGAGGGTGTATTATGATTTTACACAATCTCCAACTTTG
AACATTAATTTCTTTTGCTATAAATTATCAACTACAAAAACAATATCATATGAGAGTATTTTTTAAATTTGAATCT
AATGATACTACATGTGTAGCATTAACAACATATTTTTGTTTAATTATCAGTCAAAATTTTAAAATGGTGATTTTA
CAGAAAACAACGATGTCTTATATATATATATATATATATAAACTAACTAACTAACTAGCATCATTACTACAAAGCT
TGTCCAAGGTTGAACAAAATCATCTCAAAAGCTCTAACCATCATATCATTCCCTCAAAAGGCACAAATCATTCC
TTGCTAGACTGCTTC**CTATGAGTAAATTACATCGAGCAG(1F)**TAATGTTTATCGGTTATCTCTATGCACAACCT
AATAATACATCATGCGAGTCCACAACCTTTTTGCCTCCTGCCAATCTATAGTATTACATAATTATTAGAACATAG
CATATGCCATTGTTTTTAAAATTCTTCCTAAATATTTTAACAAATCAATTCTAAATATGTTAGAACATCTATTATAA
TGTTTCAACAATGCACATAGTTTCATCTAGTTTCCTTATGCCTCTCCGTAGTCGGATCCTTGATTTGTCTTACGA
CATTAACAATGTGAAGTGCAAAGGTGTTCCTAGGGAGAAAACAAGTCCACATAATCTAGCGTCTATGAAAGA
GATGTTTATCTAGTTAGTAACCAATAATTCTGATATTGGTTTTCCCATGGGTGTGATGGATATTCATTGCAACTT
ACAGGCAAATAATATTATGCATTTTCTTATTTCGAAAAGATATCGTACGGATGAAAGTACATGCATACACTTTTA
CACAATATTATACAAAATTAGGTTTGCTTTTCTTATCTTCAGTCTTATAATATAAACATGCAAGCATATACAAAAT
ATTATCTCAAAAATTTATAATCAACTTATGTCAAACATGCAAGCAAGGATGTTGACAAGCTTTAAAGTTTCTTT
TTAAAAAGGTGACACTTATCTGAAATGATCCCAATTATCTCATTAGTTTGGATACTATATTACTCCTAAGGCATG
CCAAATTTATAATTAACCTGACGGGTTAATTTTTTACATTATGATTGTAGATATCATGAAAACATGTGCTTAGAT
TATTATTAACCTAGAAAAATATGGGTTTCACTATTTTAGTTCATAGCGTACCTGTAGTTCACACAAAAAGTAAAT
AGTATTGAGGCAACTTTCAAAGTTTAGATATAAAGAGAAGACATATGCAAGATATTTTAAAATATAACCTTTTT
TTGGCCAAAAAAGCACTGTTATTCTATGGGATCGCCAATCACACTTAACCATGCAAATATGCAATTTGCATTGC
CATA**AGATATTTGCACTACCATTTGTCG(2F)**CATATTTCTTACCTAATTTGCATCTCTACCTTTGGACCACAAAC
TCTTAGTCTACAACTGCACATACCACTATCAAGCATGCCAGAAGGTATGATGTAAGGACTATCGCAAATCACG
TCTATAGTAGAAAAAAAAATCATGCCATATAAGGTTCATGGAGCACATGAAGGCAACCATGATATGGATGATG
ATGATGACCATTAAATCTTCTT**TAGGGATTTGGTGTGTAC(1R)**TAGTAGTACGATTTAGGTATTATTAGTCTTG
CTTCATGGAAGTACAATTTAATGTCAACATCACCATAGATCTGCTAATCAACTAGAGCAAGGCTGATAATATTT
TCAAAATAATTATGGGCACAAATAGATGACGAACTCTTAGATGAACTTAAGGGGATTGTGGAAGACAACAAT
TGTCAGACGTTGATGAAGATCCATGCATTTGTTTTTATATGTATCTTGATCATTCATCTATACTAATATAAAAGAT
GCTAGCGGTGGAATCTATCATCACCACCACCCAACTACGATGTTAGACCCGCAAACAGTGAGTCTTTCCGTAT
TCCCCAACCCCTACAAGCCATAGACCCTAAGACCTCTAGCTCAATTGGCACAACAAAAGTAACATGATAATAA
TATATTAGTCAATACAAATAACATTTTTAGGGACTTGAAAATATATACAAGGAGCCATTTTTTCTTAAAAGGCA
TGTGCATTATGCTATTATGATTGATAAGCATAATTGATATGCCACACATGAAGTATTTTTCATACGGCCTAATGAT
GGATTAATACTAACCAAATAAATTTTGTAAATTATGATTATTAATTATATAATTGTCCTTATTAGCTATCTCGCGG

AAATTCCTATGTTTCAAAAAATCTGCATCACCAAACTCTTTTGAGGGACCAAATATGCATAACATCTGACTTAT
ACTTTTGTCATTTCTCCATGTGAAAGTGTTTAAGTCAAAAGTACTATAAGAACATGTGGATTTATGTAGGCCGC
TATCTATCTAGACTCTCAATGTGAAATTAATCACACAAACACCAGAGGC**CATATGGGTCTCTACTACCAAG(3F**
)CTGGGTATCACATTCACACCCAGTGAGCTCACACCTTTTGTCTATCTGAATCAAACTTTTTTTTTACCATAATG
TGTTCTTCCTACTATATACCAAGTATATTGTTCTAATCTTAGATGGAAAAATAAATAGAAAAATAATCTAAAAAG
ATGGATCCTCCACCTTCATTAAGAGTAATGAACTAGGTGCTGATACAATAAAA**AGAGTTGACTAGATTAACCG**
G(2R)AATAGAGTCTAGTCTCTAGTTGCCTAAGAGCAACCCATATTTATCCATTCCAAAGTAAATCTACTGCCTG
AGACCATCTCTCTCCCAAGAACATATGCACCTGAATGTCCAGAAGACCAAAACCAGCCCATGGTCCATGGTA
CACTATTAGGCTATATAATATCGATCGTCCCAAATGCACATGTTTGAGTAGAATTTTTTTATTTAGACCTTTTTAA
TAAATAATTTTAATAATAGACCTCATCGAAACTTATTTTCAAAAATGAGCCTTTTGGCTATGCCGTTGGAACTG
GCGTGGCCAAACTCTTTTGCCGCACCGTTGGTCCCAGCGTGGCACCTGCTACCTCATAACTGTCATCGTGTT
GATTAAGTGAACGCCGACGTACCACTTCCTGTCACGCCAGGCTCATTAGTGTTGCAGCTGCCAATTGCCACG
CCTGGCTCGATGGCATGGCAGTGTCCTCTCTCAATTCTTCCTCCCATCCTGTGAGAAAGTAATTTTCATTGGG
AGATCGATATTGTTAGGATGTATTTTCATTATTTTGTTGGCCATTGTGGTTTATAGATGAACATGACAAGTTTAT
AGTTACAAATTTGGCACATAACGAGTAAAGATCAGATGAAGTATTAGCGGTATTTGTGAGGTATTGTTAGAAA
ATTCCACAATAACGAATGGTTTGGATGGTCCATTTTAGAAGTGTTTTGATGGACTTGATTTATGTGTTAAAGAT
ATGAATGGTTCAAGCATTGTTGTTTTT**GTCTTTAATACAATTTAGATGGGT(4F)**GGAAATGACTAAATCCCTC
GTAAGCAACCATCGGTTGGTGTCATAGGAAGAGAAATTTCTAGCACACGTTGTAACAACCACATCCATAGAA
CAAACTTGTGATCTTGAAACGAATGCAACATACGTCGACCACTTCAACTCACATGATATGAGCATCGCCACAA
AGTATTTCCTTGATCCAATCCCTGCAACCTTCCATTCTTGGCATGTCCATA**GGTTCGACATGTTGCACTTTA(3R**
)GATCAGACCGGATCACATGATTCTACCCATATAGGATTGGTCGTAAACACTAACATGTCTACTTCCATACCATC
AAACTTTCTATTTGCACTAATGGTTCAACCGAAATATACACGCACCCCAGGATCTATCTACATGCATAATATTCA
CATGCAAATCAAATACTCATTATAGAACCACGTGACCGAGCAAGCAAACGACTTCTATAAGGAAATAAGCAC
TGCATGCACGAACCTATCATCCCTACATAATCAAAATACCCTACAAATATCATGTCCATTCTATCTCATGTATAAC
TCAAATTCTATACATCGCCGAGCCTGGCATGGCAGTTACCACGTGGGCTACCTCTCCACCAAGTCGGAACGA
CGACAGCCACGGTGGAACATGTTGCCACGCCAGGACCGGTGACGTGGCAGTATTCTTTGCCATGCCATATAC
GTGGCGTGGCCAAAAGGTTTATTTTTGAAAATAAGTTTTGGCGTGGTCTATTATTAAAATTATTTTTAAAAAA
GGTCTAAATAATAAAAAATTGTCATGTAATGAAGGTTTGTACTCTAATGCCATGTTATACCCTAGCTTTAATACC
CGCATTATTCTAAATTTTAGGAAAAGCAAACTCCTGTACATTCATAATTCTACATACACTTTAATCCACAAGATA
TGTAAAATGGTTAACCCAAAAATTCTATGGTTAGAACGCATGTATTTTATATGTTGATACCTACCTATTAAGACT
CCACCCAGACTTATTCTACCATCACCACACT**ATCGGCCAAACTAGGGAT(5F)**GGCAATCGGGCGCGACGGG
CACGGGTAGTGCCTACCCATACCCATGCCCGTGAAATTATTTGTGCCCGTGGGTATGCCTGTTACTACATGAC
GGTCAAGGATTGTTGCCCATGCCCATCGCCCGCAGGCGCCTTATGCCTGCGGGCGTGCCCGTTTACCCGCCA
CAGCAAAATCAGTGGAACAAAAAGTCTACAAGCTTCTAAGTTCGAATCGAACTTAAAACATCAAATATTCTCT
GCCTCGATGTGGTGTCT**CTCCTTAGGCGGCGATAGAG(4R)**GAAGGGGGAGAGAAATAATAAAGGAAATAA
AACGGAAACCGTAGAAAACAAAGAGGCTTATGTATTCTAGTGGATTTTACTCCTAGCCTACATGTAAGTTAGA
TTTCGCGGGTAAACGGGCAAAGCGGGCATGGGTAGTGATTACCTATACCATGCCCATTTACCCTTCACCATAG
CTTTTGATCTAATAAGAAACCCATGGGTATAAAATGGAGAACAAACCCAACCCTAATAGGGTTTTTACCCACG
AGTAAACGGGCAAACGGGCACAATTGCCATCCCTAGGCCAAACATGTGTCCACACCTTCACTTACATCGTGG
ACCAACGTGTGGTGCTGTGGTTCTTGGATGTAAGATTTATTTACCAATTGCCCCACGCGGATCATTAGAGATA
AACATATTATTTTTTTCTTTCTACCACTTATTCTAGCCTTATTCTATCATCGCTTTGGGCCCATGAAGCGAAGGG
AATGAGCTCAACTGCTTCTCTTTTACACTTCTTTATTTCTCTGTGCCCTTCCGCATGCGCTTCGTATGTCATTGG
CACTTTGATCTCTTATTTCTTCATTGGAGGTTTTGGATGAACTTCGTCGTTCTTTCCCAACAAAAATGGAAAG
GGTTGTATCACATCAAATGTCGATTTGTTTTCCGTCCCAAATGAGATGGGGAACTAGTCACCTTTATCTCTTCA

TCTCTATGAAAGTCTTTATGAAAGGAATCGAGGTCTGGCCCGACCCGGTTCACGTCATTCCAACAACTGA**CG**
AGGAGCACCTTAGTGTACG(6F)ATCACGTCGATCAAATACCTATGTCATTTGTCCTAGAACTATTTTGATATAA
TGTATATCTATTTCGCGAGCACTCAACATTAAGTGCCTATCGTACTATCCATATAATGCCATTATGTCCTTGTTTA
GTTTGAATTTCCATGAAATGCTATATGGAGTAATACAAAAAAAAGAGCAAATATTACAAGTATCGCAAATGAG
ATTATCTTCAAAAAAAAAAAAAATTTGACCAAAATTTCTTAGAAATAATTGTCAAAAAGATATTGGA**GTGTATT**
GCGCTGTTGGT(5R)GTAAAAAACTAGACAACGATGCCAATGAGGATGCACTTGAGTATAACAACTTTAAAA
ATAAGTGTATTGATCTGATTGATTACTTAGTTCGTAGGTGCATTCTTGGACATGATTTTTTTTTCCATTAGACAC
ATTAATTGCGTGAAACTTCCTTACAAATTTTGCAAAAAGTAATTTAAAGTAAAAGTTTCAAGAAGTGCTTTGA
GTTAATTTGTATTATACTCCCTCCGCACTCGTAAAAGAAGTCATTTTGGACAGCGACACGGTCTACAAAACGC
AACTTTGACTTCTTATTTCTATAAAAATATTTATTGAAGAGTGATATATGTATAATTTTATGAAAGTATTTTTCAA
GACAAATCTATTCATGTAATTTTTATATTTTCAAACTCAACAACTTGTGAGTTATTCATGATATATATTTTCAAGG
TTTAACTTAAACATTGTTCTAAACGACTTCCTTTATGAATACGGAGGGAGTATGCATTATTATTGTACTGGAGG
TCATGATCTAGCCATATTAAGGACATTGTTTGGCACGGAATACCATTATTAAACTGAAATTGTAGTTACATTGA
AAAAAAGTAACAACACATAAATAGATATAGATGATATCGTCCAAAACATCAAGGACAAAAAAGATTAGTAGTT
TTCCACAAAAAAAAAAAGTATGGAAGTTACATTTTGCCCCTTGATTTAGCCAAAAACGCACATGGCACCTGGT
TTGTTCAAGAACACACACATAGCCAAGTCACATTTAGCTAGTTGTTGAAATGAATGAATTA**AACCCGCTACCA**
AATTACCAC(7F)ACCGACATAGTAAACTTTCAAGTTTTTGCAATCAATGTACCCACCTGTAATTTTTTTATTATC
CAGAGTGTAATCACTCCTTATTTAAGTAGGACAAACAAATACATTAAACATTTTTTAATGTAACTATATTGCCAT
TAGAGAAGTGCTACACTTCCAAAACAAAAATTCTTACAAATGTTTTACAAACGATGATGTAAGATATTGTGAT
TTAATATTGATTTTCTATAACTTT**GGACTAATTAGATCAAACAGT(6R)**TAAGAAATTTGTTTTTAAGAATTTTG
CATGAACTTTTTAGCATGTCCAACATTGCTCTTGTTGTTACAAAGGTTAAATCTTGTTTGCAGAGGCATTAATA
ATATTACGGAGATACTGAGGTTATGTTCGGGAGTGAGTCCCTAGCATGCAAAACGAAGCATCGTTTAGCACA
TGATTAATTAAGTATTAACTAAAATAAATTTTAAAATGGATTAATATGATTTTTAAAGTAACTTTTATATAGAAAT
TTTTTGCAAAAAAACACGCTGTTTAGCAGTTTGAAAAGCGTGTGCGCGAAAAACGAGAGAGATGAATTGG
GAAAGTAGAGATAAGAACTCAGCCTAAATAGTTTCTTTGAGTTAGTATAACACATGTTCAATAAGTGCTGTTT
AGCAGTTTGAAAAGCGTGTGCGCGAAAAACGAGAGAGATGAATTGGGAAAGTAGAGATAAGAACTCAGC
CTAAATAGTTTCTTTGAGTTATTATAACACATGTTCAATAAGTTAATTGTATACAAGTTTTCATTGGTGCCAAAA
ACTGGTATAACAATGACAATAAAAACGTATGTTATTTTTCACTAGAACAACATTGTAATTACATGTGTTGACCT
ATACAATGTTTGGTTTGCCATGATTACGATAATATTAGAAAGCTCCTAACAAATATAAAACTTGCCCATTTTCTT
ATCCAATGATGAAATGAACTATTTCATTATCAAATTTTAAATGCAAACAAAGATAATCTTAATTCGATAGAACTT
TCACATAAGAAAAAGTTATATGCCAACTTCCTAATGAGTTTTTGAGTAATCCCTTGCGTAGT**TCCATACTGTGC**
TTTTAGCCAC(8F)ATTGTTATCAACATATTGGTTGAATTTTGGGGCATGCAAACTTATACAATGACCCTCGAAT
TACACTTTTTGAATTGTAGGTATGAGGTGTTTGGGGGCATATTATCAACGAAACATATCCTCACTAGGTAGAC
ATGATAATTACATGATTAATTTACAAATGACTATCATAAATATAT**GGAATCTCAAAAGTCCACTTG(7R)**GGACA
CGAAATCTAAATGATAACATGAAAGATTGTTAATAGAGACATGTTAGTCCAAATTGTTTATACTTTCGCTACTC
ATATATTAATTGAAATAAATATTTAAATGTGCAAGTAAGAGAACATCCATGCATTGCAATGAATCTCAAAAGGT
GCATAGTAATTTTGAAGTCTATTTAAATTCTAAACAAACCACAATTTAAAAATAGCCAAACTATGTGACAAGAT
ATACAAATCATTCATTAAAGAGGTGGCACACTAGAGCCACCACTAGTCATTTTATATAGAAGAAATAAATTTAG
ATTGTCCAACAATACCTTTTTTTTTTCTAAAACCTTATTCCATCATATATTTTTTTAAGAACTGAAACTCCGTTGT
AATGTAGACATTTTACCATTCAAAGAAATGCTAATATGGTTGGGAATTATTTTATACTTGCATTAGATCATTTAT
TATAACCTGGATGTGAGTATCCATAGGATTCGTGAGTTCTAATTAACAATACTGCGAAATATGTAACACTTTTA
ACATAACATTCACGTTATAATGATGTTAGTTAAAACTTTATTAACATCCAAAACAATT**TTTTCATTGGACAAATA**
AATGCTC(9F)ACGAGGAAATAGATGTTATCTCAAATTGGTATGAAGATAAAGAATGGTTGACTAAGGAATCTA
CTAAAATACCTTTTTTTTTCACTGTGTAACTTGTTGGTTTGCTTTACACTAGTACTTTATTTTTTGGGTTCTTTTG

TATTTCTTATAGCAATGTCATTCTTGTGCTTATCTCCTTTGCTGTGTTTTAAACATAAACATCATGTTTTGCATTG
ATTTCCCTGTGTTGCTAAATATGTGTGTTTTGATATTTATCATGTTCTATTATTTAAATATGCATGAATTGAAGTAT
ACTCATATATCCTATTTCCACAATACATACTTTAGTTTGTTACCAACTATGCTGAATTCGGTGCTCACATTTATTA
TCATCATGTTATGGTTTGATACAATTTGACAGTTTCAAGTTAAATTGGGTCAAAA**GATGTTCATCGGAAAATT**
CAAC(8R)TTGGGTAAATATTTCATCAGATTGACTTCATGCTCTGATCTTCGTGGAAATAGAGAAGGATCTGCT
GACTGATTTAGCTTTTGAACAAGTGCAACTTGGTGCATCGACAAGATTTTGCAGGAAATTTACTACATGGGG
TGGCCATCATACTGAAAATAATTTATATAAAAATTTATGTTTCATCATTATTATATTCATGTGATTGTCTTATACAT
AACTATTTGGATTCATTCAAACCCTACTCTCTTTTGTAAACTTGTATTTTGCAACAATATTATCATTGGTACTTAG
ATGAAACTTGGTTAACAATATAATCATTAGTTCTTAACTTTTTAAGTTGACTTTTTGAATATCCGTCCTTGTATT
GTTTTAGCTATGTTTTATTAATAGTTGTGTTTGATCTTTTTTGTGGAGTTGGTGCCTTATTGTTTTGTTTACATTT
CTAGATATATTTGTCCTAGGATTGGTGCTCTTATGCATACTCATGATGAGGTACTCATTTAATGAAAAGAAATAA
AGATTGTACCTCATGTTCCAAAACAACATATTCA**CAATTCATTATCATGGAAGTC(10F)**TCATCCAAGAACCTT
TTTTAACTGGCGTTTCTTAAATTTTGCAAAAACTCTGACTTTGAATTAAGATGGATAAGTCCCATGATCAACAT
AGCTTTCAATTCTATATAGTAGTTGTTATTTTCTAAATTAGTCAACATCTCCTAATCTTATACATTGTACTACTTAC
ATTTTGCATGTTTATCTTCATGATAGTTCTTAAATTTCTATGACATGTATGTAATTTACCTTTAGGTTTTATGAA**A**
AAGGTCGATGACAATGA(9R)TATGAAATTAGACTAAGGACCGATATTTCGCTGTCAAGAATTGACATGCTAA
ATCATTTGTGCCATAAAATCCAAATTGAAAACTCACTTGTGTTAACATTATTGGTCAAAACAAATTATATATCTA
TGTTCTAATATCATGAAAAATGTAGAAAAAAAAGTTCTTAAAGCACTTCGAAGCTTCCTATGCAAACTGCTCT
CAACTAGTGCCTTCAGAATATTTTAAAATAGCAACTCCTACATAGCCTCATTCAAATCAAAGTTTCAATCACAC
TATTGATATTACTTTGAGCTATTTACATACTAGATGTTTATATTTGTTATATTAATAGAGGTTCATGTTGGTACTTC
TAAGAAATTGGGTCATATTTAACATGCTTAACCTCATCACCTTCAAACTATGTTTTAGATTTGTTATGTAAAACT
AGTTCATATTTATTATACTAATAGAGGGTACATGCAACAAATATGACACATATTTAATGGTTAAAACCTTCTCCA
TTACGGAAACCGATTCAAATGTGTCATAGTTGGTCTCACAACAAAAAAAATAGGTTATACTAGTCATGAAAAT
ATACAAAATCAAGTTAGAACATAATAGCGTAATCTAGGAATTAAAAGTGCATAAAAGGTGGCTTTTGATAATA
CGATGGTGGAGGCTGGGCGCACATATCATGACCGGTGTTTTATGGAGAAATTTTTTATTGGGGCTTGGAATA
TTTAAAAGCAAAGGAATGGTTTCATCTTTGAAAATATTCTTCCTTCCTTGGTTTCTTGGAGACAATCCCTTAA
GGTGATCTCCTTTTGTTGTTTAGATTGAGTGAGGGTGATAAAGATGTAGTCTTAAACTGGATTAGGTTGTTGT
AATCATTTATATTTCTTTTTATTATCAATATAGTTTTTTAACAGCGGGGGGCCTCCTATGTTGTGTTCCTTTAAAA
AAAGTGCATAAAAGGAATATGTCAGGATATTATTTCCATTTTTAGTATTACTAGGCTTTTGAAAACATAAGGAA
CATTGAAAAGAAAATTGAATTAA**GTGCATGTTTAATCTATAAATTCA(10R)**AAATCGAAAGAGTTAATACATA
TCGTATAGAAGTTCAACACACCAAAGCAAAACATACTACTATGACTACTTGTTGAACTTCACAATATGCTATTC
ACAAATGGTAGTGAGATGCAAGTAGCACTCAAATTTAGCTAAAATAAAATAAAAAGAATAAGCTACAATTCTA
GTAACATAATGGTTCTTAGAAGTATTTTCATTACTATTTGCCCTACTTGAGTGATAATCATCAATAATAACTAAA
ACATGCTATCAAGATGCACCAAGGACTCATGGTGTGCAAGTGTTCAATAATTATATTCCTCTAGTGGATCAAA
AGTATGCACTTTATGAAGGATATGGGTAAAGTTATTGATTGAGTTGTTTGAAATTTCCTTTGGCATATCATTAC
TCTAGACCTTGCATGAAGAATGCGTAAAGTTTATATTTGAGTTGTTTGCCATTTCCTTTAGCGTATTTTGCTCTA
AACTTTGTAGGAAGGATGGGTAAAGTTATTGTTTCGAGTTGTTTGAAATTTCCTTTGGCATATCATTGCAAGA
CCTTGCATGAACAAAGGGTAAAGTTTTTGTTCGAGTGGTTTGCCATTTCATTCACCACATTTTGCTCTAAACT
TATGTAGGAAGGATATGGTTGGAGTTATCATTTGGGTTGTTTGCCATTTCCTTCGATGTCTCATTGCTCTAGGC
TTAGTTGACATCTTGGTAATTTGGCTCATCTTGGACTTAGCTATATATTTATTGTCATTGGAGTCATGGTTCTAG
TCATCCTAGGATCGTGGTTAGTGGCCTTGATGATCTTACTTGGTTAGTTGTGGTACTTGGTTAGTTGGTACCAC
ATTCCTTGTAGAAATTCGGTTAAAGAAACATTACTGGAGTTTATAATTCCCAAATGTAAATCATTGTGAATTATT
AATTCTCTATATACTCTAACGGGACAAAAGAAAATTATTCATACCAAATCATAGATACTATTTGTACACCTAAAA
ATATAAGATTGTAAAGACCATGTATCTCATAGTCAACAAATCATGTTCTCAATCATGTATATATGAGGACATGCA

TCTAAACTTGATTTTAACAAATTTTATTTTAAAAACAAAAAAGTAAAAAAAAAAAGGTGATTTATATTGGTAA
CACATATATCCTCTACCCTAGAGAGGCCACTATATCTTGGTTGCTATGCATTTATCTCCCTAAGACCACAATATTC
ATCCATATGAGGGGCATCCAATGAATTATCTCAATTCCTCTTGACTATGGCCTTATTAACTGCCCTACTTATAAA
GTCTCAATATGTCAACATACTTATTGGCAATCATATGGCCAACATGTTGATCAAGTGTTTGCGTAGGAGTTGAT
CCAACACTAGTATCATAGTTTTGTATAGAAGGTGAAATCTATCACCTTTTACATAGAAAATGATCCTACAGTGG
AAACCTAAAATGGTGATTTCTTGAAATGAAAACCATTAGTATGTTTTTTTTTAATATGAAAAATCATTGGTCTA
GTTTGTCGTTGCCATAACAAAAGTTCTGACAATTTTATATTAGTTCAGTGTACTATAGGTGAAACTAGTTCATA
AAAATATGCATTGTCTTGATTTCTGAATTTTGGCACCAAAAATAAGTGCACTAAAGGATTTAAGGCGTTCTATT
ACTAATTTAGAGCTTTTTGTTTCTGGAATAACCATAAACATGCAGTTTAAATCAGAGATTCAAAAGATTGAGC
ATTAACACAATAGAGCTCTTTGTAAATATTTCATTTTGATCATAAATCTAACTCTTAGTGAATTATAGGAGAAGA
CGGCATGAACTCAGCATTTTTATTGTCCCCATTGTTTATAGTTCCTTTTGGTATTTAGCCAAAGCCTTGAAGTT
GAATCATATCGACAGCCTAGGAGGATATTTTATCTAACTCTTAACTGCCTCGTTTTCTTTTTCAGCATTGAAGA
TACCTATGATCGCTATCAGCAATTCGCTGGAGCCAGGAGAGACCTGAACGAAGGAAGTACAAGCATCAACA
GTGTAAAATCCCCGTGAAACCTTTTTTTAATCTGTCCATAGATTTATAGCGTGTTGCCGCCAGGTTTGATCATT
TTGTTGTTTTTGCAACCAACTTCAACATCCCATTATTATAAGGATTGAATGTGTGGCTGCATGTTGCACAGGAT
TGGTAAAATTAAAGCAAGAGTGTTTTTTTTTTTTTGAACTAAAACTACTTCAGGAAGGCCCCGCAGTTAATAT
ATTAAATTAAAGGAGAGGTGTTTTTTTTTTTTTGCGGGGAAAGGAGAGGTGTTACATACAAAGAAATGCTTA
TGTTTATATCTGGAGAACCATCTAAATTAGACTAGGGGTACATTTTGCATCCAAGATGACCAAATTGTATGGCT
TTGCCTGCTCTCGTATATATACCCCTGACAGGTATTTTGATTCTGTACCAGGATGAAAATGCAAGTATACACTAC
AGGCTTAGGGACATAACGGCCTGGTAACACTCATGAGTTTTGACTACACGTCCCAAATCTTTATTGACTTTGC
TCAACAGAAATCTGAGCATTCTAGCCAACCTGCAGGTCTCTCCAAAACAATGCTGACGAGTCGGATGCTAAT
CAGCTAGAGAAACTGGAGAAACTGCTGACAAATGCTTTGAGGGATACGAAATCAAAGAAGGTACATTGTAA
TGTCACTGTTTCCACCAAATATTTCAACCTGGTTTAGAAAAAAGGCTAGGAGTAGAAGATGTGATTATTTTGG
TAGCTGGTTCAGCATCACATTTTTCCTGGAGCTAGACATTAGTTCTGATGGTTTACAGATTCTGTTTTTCCTAT
TGGTATTATATTGCAAATATCCAGTGGACCAGTGGTGTGGAGTTCATGTAGCATATTTTTAGTCAGCCAGAGA
AAAGGGATTCAAAGAAATACCAATGTGGCTGTCTTGAGAAAATGGGTGCTCAAAAAGTGGCAGCATCTCTC
CTTGCATGTGGCTGTCTTGATAATTTCAGATTTTTCAGTGTGACACACCAGGATATATTATATAACTACTATGTA
GTCGTCCTTAAGCACAGATAACTATATTCGTGGATATCATATAATCAATTGAAATGAACTGGGAGATCTCTCTTT
TTTTTGAGGGGACAACTGGGAGATCTATCATCGATGCTGAGATTGGTGTGCTTCATTCAAAAGCGCTGCGAG
AAAACTCTGTATGCGGGCAATACTATTGCATAAACTGTTGATGTCAAAAATAACTGATATTTTCTGGTTGTACT
CTAATCAAGCTTCTTGTGATGTTTTTCTTTTTTGAAGACTTCAACTGAAGGCCGTGAAACCGGCCCACCTCTC
CGTTCTGCCGTTTTAGTAAGCCTAGTAACAGAACAGGGATATAATGATTGGCACCCATGGATTCAGTCCTGAT
AGTATTGCAATATTGTCAGCTACCTGAAATGACAACTAAAATGTTCAGTTCATGATAACAGAACATGTCTGTCA
GTTACCTGAAGATGTTGATAATTCGTAAATGTGGTTTTTTTTTGTCCCTGTTATTTGTCCGTTTACCTTTCCTTG
TTTTGGATTGCAGATGTTGGCAAAACAAAATGGTGAAGGGAGTAGGAGCAGAGCAAACTCCAGTGGCTCT
AGGGGGCAGGAGGAAGTGCATGAGCAGGAAGCACAGGTGTCCTGTGCATAAGTTTGGCAGTGCATGCAC
CTCCCAAATTAAAAAGCTAATAAAAAAAAAGAGAAAATGGTGCATGCTCTGTTTGCTGTCAGTTTCTCCCACT
GATTGATCCCAGGTAGAGAAAAGAGTTGCCATGGCCTCTGGCTCTGCTTGTTAGATCAAGGGGGGGAGATG
GAATAAAAGGATGGGGAAGTATTCAGGTTCTTGTAATGAAGACCTATGCTGTGGTGTTTTAGCAATGTTTGA
TGTGTGTGCTACCTGTGTTTGACTGGTTGTGTTGCTTGATGTGAGTCATCTAACCATGATACTGAAAGCCTGA
TAATTGCACTGACTTGTGTATGGAGTTTGTTTCAACCTATTGGTATGAATCTGTTTGATTCTGTAGGAACTGTG
GTTTGCTGTTTGCC
